# Long-Term Maintenance of Human Pluripotent Stem Cells on cRGDfK-Presenting Synthetic Surfaces

**DOI:** 10.1038/s41598-018-19209-0

**Published:** 2018-01-15

**Authors:** Jack W. Lambshead, Laurence Meagher, Jacob Goodwin, Tanya Labonne, Elizabeth Ng, Andrew Elefanty, Edouard Stanley, Carmel M. O’Brien, Andrew L. Laslett

**Affiliations:** 1 CSIRO Manufacturing, Clayton, Victoria, 3168 Australia; 2 Australian Regenerative Medicine Institute, Monash University, Victoria, 3800 Australia; 3 Monash Institute of Medical Engineering, Monash University, Victoria, 3800 Australia; 4 Murdoch Children’s Research Institute, The Royal Children’s Hospital, Victoria, 3052 Australia

## Abstract

Synthetic human pluripotent stem cell (hPSC) culture surfaces with defined physical and chemical properties will facilitate improved research and therapeutic applications of hPSCs. In this study, synthetic surfaces for hPSC culture in E8 medium were produced for screening by modifying two polymer brush coatings [poly(acrylamide*-co-*acrylic acid) (PAAA) and poly(acrylamide*-co-*propargyl acrylamide) (PAPA)] to present single peptides. Adhesion of hPSC colonies was more consistently observed on surfaces modified with cRGDfK compared to surfaces modified with other peptide sequences tested. PAPA-coated polystyrene flasks with coupled cRGDfK (cRGDfK-PAPA) were then used for long-term studies of three hPSC lines (H9, hiPS-NHF1.3, Genea-02). Cell lines maintained for ten passages on cRGDfK-PAPA were assessed for colony morphology, proliferation rate, maintenance of OCT4 expression, cell viability at harvest, teratoma formation potential, and global gene expression as assessed by the PluriTest™ assay. cRGDfK-PAPA and control cultures maintained on Geltrex™ produced comparable results in most assays. No karyotypic abnormalities were detected in cultures maintained on cRGDfK-PAPA, while abnormalities were detected in cultures maintained on Geltrex™, StemAdhere™ or Synthemax™. This is the first report of long term maintenance of hPSC cultures on the scalable, stable, and cost-effective cRGDfK-PAPA coating.

## Introduction

Human pluripotent stem cells (hPSCs) show potential for drug discovery, studying disease mechanisms, and for clinical applications including the generation of differentiated cell types for transplantation. hPSC cultures were originally maintained in co-culture with mitotically inactivated feeder cells. Although surfaces coated with feeder cells can provide the necessary supporting factors for hPSC maintenance, their use inherently results in variable culture conditions. A number of synthetic hPSC culture surfaces have since become commercially available, yet they are not commonly used. The widespread use of synthetic, chemically defined culture conditions would improve reliability in cell manufacture, reduce inter-laboratory variation in hPSC cultures, improve the consistency of experimental results and facilitate the derivation and application of clinical grade hPSC lines. This paper describes the screening of synthetic surfaces for adhesion and maintenance of hPSC. Synthetic peptide-presenting polymer coatings, able to support adhesion and maintenance of hPSC cultures, were used to screen for peptide ligands that were individually capable of mediating hPSC adhesion. The polymers poly(acrylamide*-co-*acrylic acid) (PAAA) and poly(acrylamide*-co-*propargyl acrylamide) (PAPA) were modified to present peptides with previously reported roles in cell adhesion and then subjected to a hPSC adhesion assay. Long term hPSC culture studies were then performed, comparing the lead surface to commercially available, synthetic hPSC culture surfaces (StemAdhere™ and Synthemax™) and to Geltrex™-coated surfaces. The lead surface presented cRGDfK, a cyclic peptide that has been optimised as a potent and selective inhibitor of the αvβ3 integrin, which is able to bind both αvβ3 and αvβ5 integrin^1^.

In this study we used a type of initiator-free, surface-initiated polymer grafting method that is relatively unknown^2–4^, but which can be broadly applied to polymer, organic and inorganic surfaces^3^. For the coatings presented here, a methodology was optimised which relied on multiple passes of the multiwell plates or flasks beneath a high intensity UV light in the presence of monomer solution and in the absence of oxygen. This method produces a surface coating, the properties of which can be readily modified and optimised for a variety of applications, including *in vitro* expansion of pluripotent stem cells as presented here.

## Results

An OCT4-mCherry reporter cell line was generated from the human embryonic stem cell line H9 to screen PAAA and PAPA coatings modified with test peptides for adhesion and short-term maintenance of hPSCs. Briefly, the copolymer coatings were prepared using an initiator-free UV-activated surface grafting method^5,6^ that prepared surface-initiated polymer brush coatings, to which peptides were chemically coupled using either water soluble carbodiimide chemistry (for lysine-terminated peptides)^7^ or Cu(I) mediated azide-alkyne Huisgen cycloaddition (for azide-terminated peptides)^8^. In our hands these surfaces were also able to maintain adherent cultures of mesenchymal stem cells and L929 fibroblasts (data not shown). After identification of the lead hPSC adhesion peptide and optimisation of the substrate coating (data not shown), three hPSC lines were maintained on the lead surface (PAPA-cRGDfK) for ten passages and compared to cultures maintained in parallel on the commercially available synthetic culture surfaces Synthemax™ and StemAdhere™ and to control cultures maintained on Geltrex™. A schematic of the preparation of the PAAA and PAPA surfaces bound to cRGDfK including chemical structures is shown in Supplementary Figure S13.

### Derivation of H9-OCT4^*2AChryIM/w*^ reporter cell line

**Figure 1:**
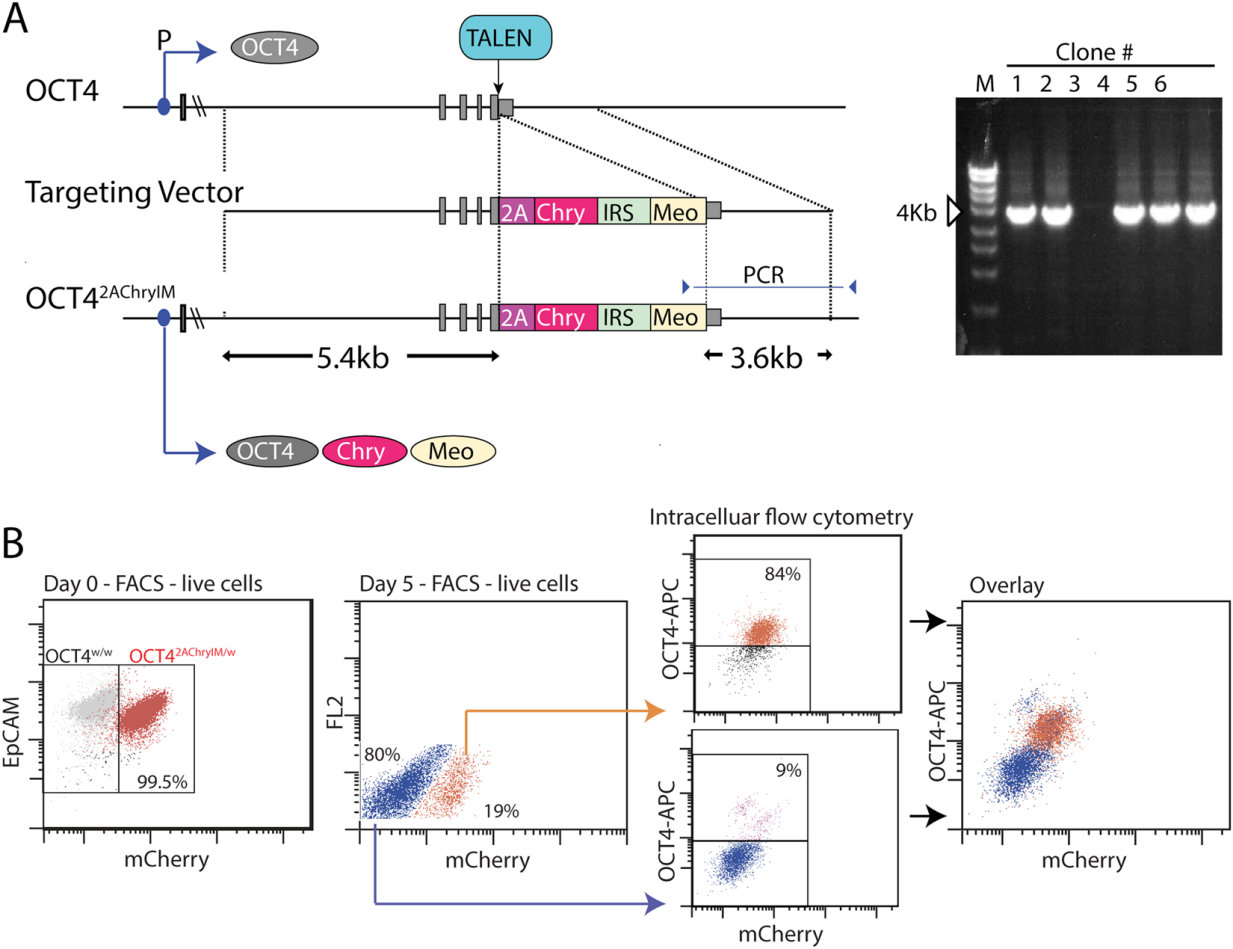
Characterisation of H9-*OCT4*^*2AChryIM/w*^ human embryonic stem cells (hESCs). (**A**) Schematic representation of the targeting strategy used to introduce an mCherry reporter gene in place of the stop codon of the endogenous OCT4 locus. The upper line shows the wild type OCT4 locus with exons marked in grey. The relative position of the OCT4 promoter (P) and the point within the 3′ UTR against which specific TALENs were directed is indicated. The targeting vector (middle line) included a 5.4 kb 5′ homology arm that joined sequences encoding a T2A peptide (2A) and mCherry (Chry) in frame with the OCT4 coding sequences. Selection of correctly targeted clones was facilitated by an internal ribosomal entry site (IRES) preceding a Neomycin resistance gene optimised for expression in mammalian cells (Meo). The three translation products of the targeted allele are shown at the bottom. The gel electrophoresis image shows that the correct size fragment (3.6 kb) was detected by PCR screening in 5 of the 6 clones screened. (**B**) Validation of H9-*OCT4*^*2AChryIM/w*^ hESC reporter fidelity using intra-cellular flow cytometry for OCT4 expression. At the day of passaging from maintenance culture (day 0) 99% of undifferentiated cells were mCherry^pos^ (left panel). Following 5 days differentiation, 20% of cells continued to express mCherry. mCherry^pos^ and mCherry^neg^ cells were sorted at day 5 and each fraction stained for OCT4 protein expression using intracellular flow cytometry. This analysis showed that 84% of mCherry^pos^ cell retained OCT4 protein expression whilst only 9% of cells in the mCherry^neg^ fraction expressed OCT4. OCT4^pos^mCherry^pos^ cells could be readily distinguished from the complementary OCT4^neg^mCherry^neg^ population.

In order to monitor pluripotency, TALEN-mediated gene targeting^9^ was used to create an OCT4 reporter line in which mCherry was expressed via a T2A sequence that replaced the OCT4 stop codon (*OCT4*^*2AChryIM/w*^) (Fig. 1A). Targeted clones were identified using a PCR-based strategy and on the basis of mCherry reporter expression in undifferentiated H9 hESC line, the latter confirming correct alignment of the OCT4 and mCherry reading frames. Expression of mCherry in H9-*OCT4*^*2AChryIM/w*^ hESCs could be observed under fluorescence microscopy and readily detected using flow cytometry; intracellular flow cytometry of partially-differentiated cultures co-stained with an OCT4 antibody confirmed that mCherry expression reflected expression of the OCT4 locus (Fig. 1B).

### H9-*OCT4*^*2AChryIM/w*^ adhesion assay for screening peptide-modified polymer coatings

**Figure 2:**
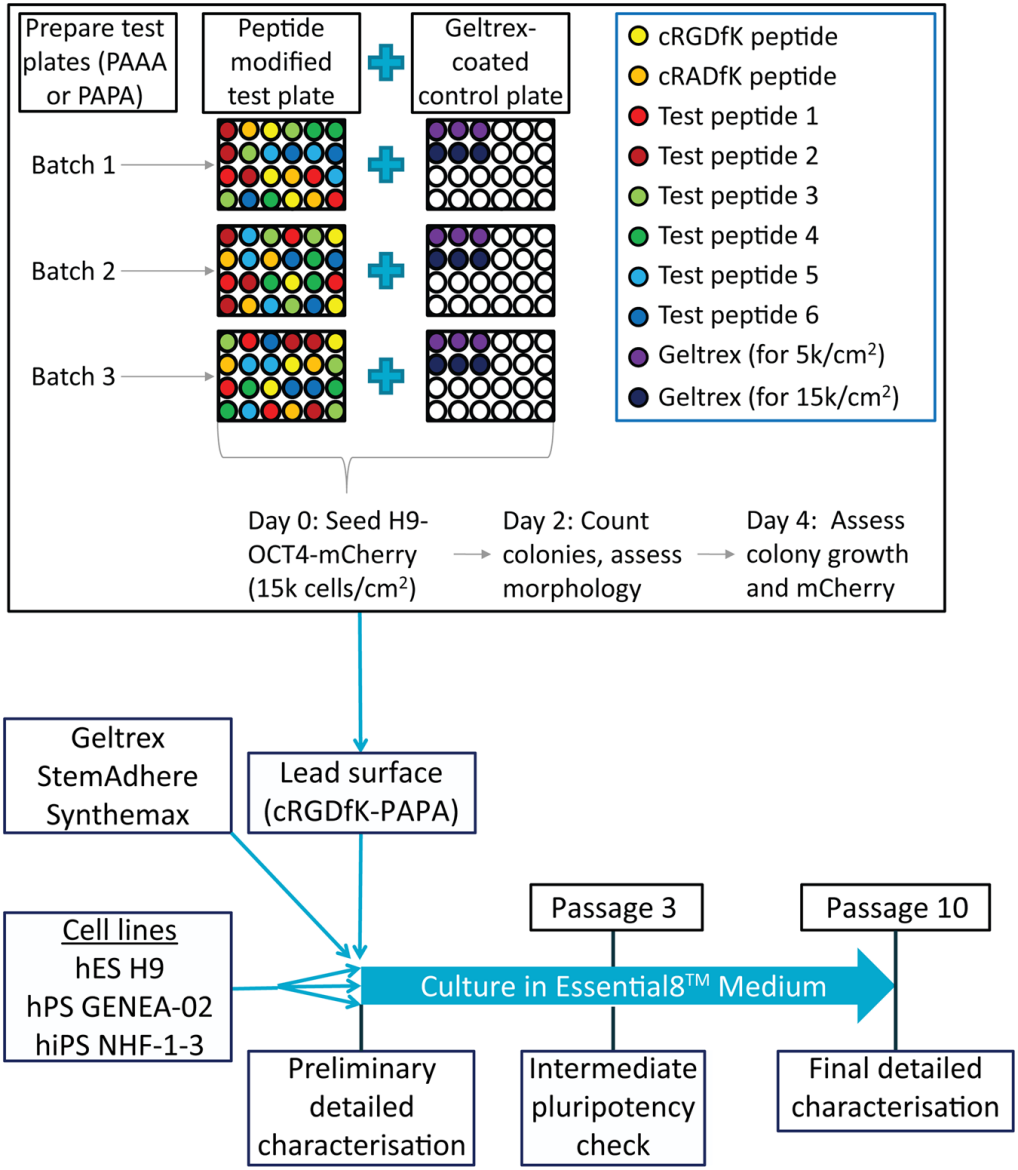
Screening approach feeding into long term experimental plan. A schematic diagram illustrates the screening process used to identify peptides that, when chemically bound to PAAA or PAPA coatings, produced a surface able to bind and maintain short-term culture of hPSCs. Three batches of peptide-coated plates were prepared and triplicate wells in randomised locations of each plate and modified with either the cRGDfK peptide, the non-binding negative control cRADfK peptide or a test peptide. Plates were seeded with H9-*OCT4*^*2AChryIM/w*^ hPSCs at a density of 15 000 cells/cm^2^. Geltrex™ (GX)-coated control wells were seeded in parallel at equal and one-third (5 000 cells/cm^2^) density. Colony number and morphology was assessed at 48 hours post-seeding and growth and maintenance of mCherry were assessed at day 4 post-seeding. HPSC cultures were then maintained for ten passages on surfaces coated with the novel candidate hPSC culture surface (PAPA-cRGDfK). hPSCs were characterised before and after this culture period, and comparisons were made to cultures maintained in parallel on Geltrex™, StemAdhere™ and Synthemax™.

The approach outlined in Fig. 2 was used to screen for hPSC adhesion to 23 peptide-modified PAAA coatings, which had been prepared using 40 passes under a high intensity UV light source (PAAA-40UV) and to 14 peptide-modified PAPA coatings that had been synthesised with 30 UV passes (PAPA-30UV)^5,6^. The full list of peptides is provided in Supporting Information Table S1, with chemical properties regarding solubility described in Supporting Information Table S2. PAPA coatings were used for lysine-containing peptides, since the presence of lysine residues would interfere with the carbodiimide coupling approach used with PAAA coatings. H9-*OCT4*^*2AChryIM/w*^ cells were observed to adhere to coatings that had been modified with the cRGDfK peptide (cRGDfK-PAAA and cRGDfK-PAPA) as well as peptides 20 (pep20-PAPA), 31 (pep31-PAPA), 34 (pep34-PAAA) and 35 (pep35-PAAA), which represented 14% (5/36) of all peptides tested. More colonies were observed to adhere to wells coated with Geltrex™ or cRGDfK-modified surfaces than to polymer-coated wells that had been modified with the other peptide (Supporting Information Figure S1).

HPSCs were often observed to form rounded clumps on pep34-PAAA or pep35-PAAA surfaces, and appeared to be only loosely attached to the surface (Supporting Information Figure S1). To further examine the pep34-PAAA and pep35-PAAA coatings, we tested thinner PAAA-25UV coatings and increased concentrations of peptide solutions up to 600 μM (Supporting Information Figure S2A). Although these conditions did slightly improve each substrates efficiency, they could not match the cRGDfK-PAAA coating (Supporting Information Figure S2B). This was repeated with another hPSC line, hiPS-NHF1.3, and found to be consistent (Supporting Information Figure S2C).

**Figure 3:**
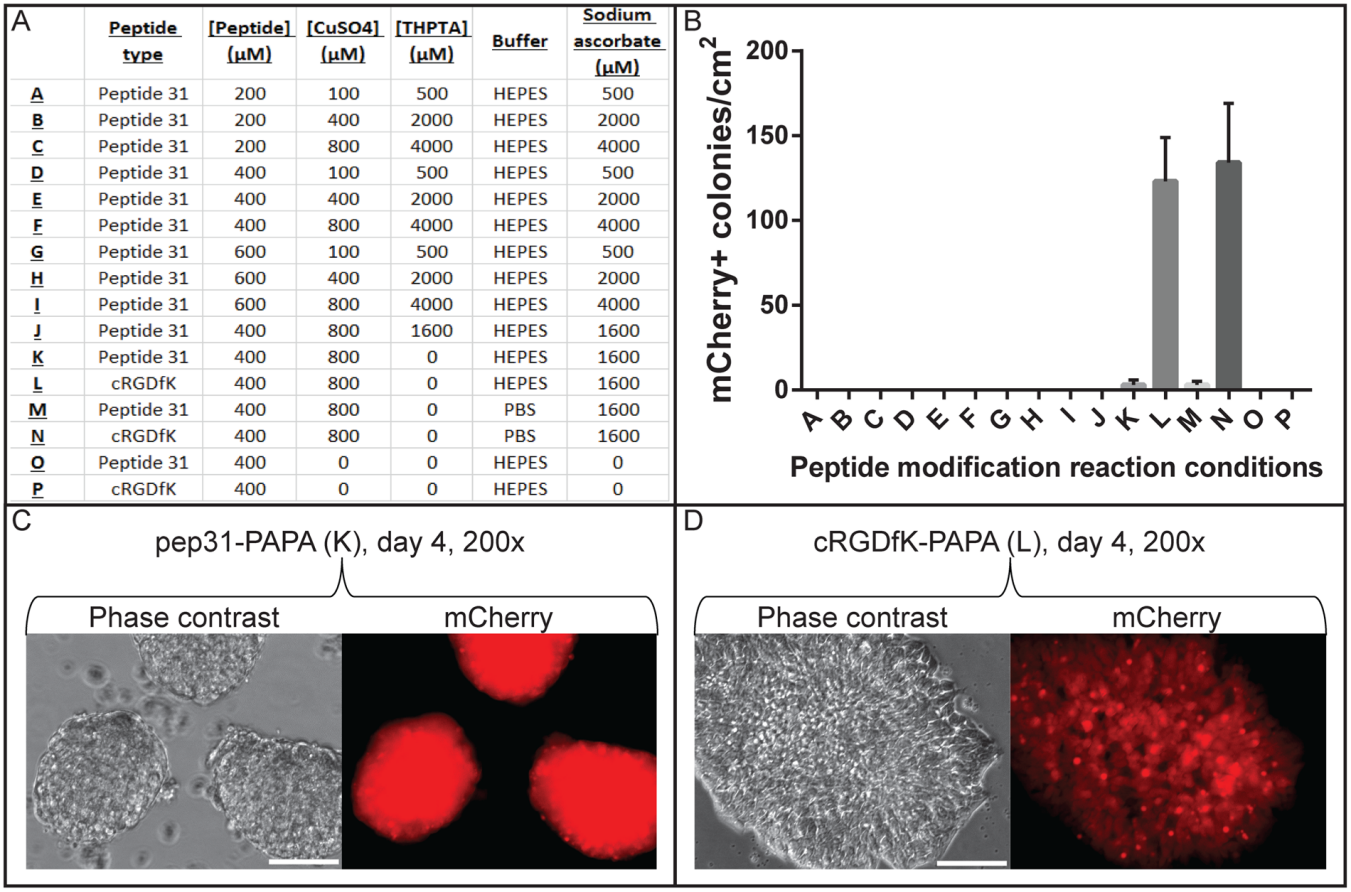
Optimisation of CuAAC peptide modification reaction conditions failed to improve H9-*OCT4*^*2AChryIM/w*^ adhesion to pep31-PAPA surfaces. (**A**) A table shows the reaction conditions that were trialled to improve cellular adhesion to pep31-PAPA surfaces. (**B**) Counts of mCherry^pos^ colony numbers on PAPA wells modified with the conditions presented in A. n = 3, Mean +/− SD Images are also presented from day 4 after seeding when (**C**) rounded colonies of H9-*OCT4*^*2AChryIM/w*^ cells were loosely attached to pep31-PAPA and (**D**) typical colonies of H9-*OCT4*^*2AChryIM/w*^ cells were attached to cRGDfK-PAPA. Scale bars represent 100 μm.

Fewer than five hPSC colonies per square centimetre were observed to bind to pep20-PAPA and pep31-PAPA coated surfaces. However, these hPSCs did not bind stably, displayed morphological differentiation and loss of mCherry (Supporting Information Figure S3). Since hPSC adhesion to surfaces modified with the HSPG-binding peptide 31 had been widely reported^10–12^, and since more H9-*OCT4*^*2AChryIM/w*^ colonies bound to PAPA coatings modified with peptide 31 than to those modified with any other non-cRGDfK test peptide, the CuAAC peptide modification reaction conditions were further optimised to improve hPSC adhesion to pep31-PAPA coatings (Fig. 3A,B). Introducing the copper ligand 3,3′,3′′-(4,4′,4′′-(nitrilotris(methylene))tris(1H-1,2,3-triazole-4,1-diyl))tris(propan-1-ol) (THPTA) or increasing the concentrations of CuSO_4_, sodium ascorbate or peptide in the reaction failed to improve hPSC adhesion; only loosely-attached rounded clumps were observed (Fig. 3C). Meanwhile, control cRGDfK-PAPA wells consistently bound 100–150 flat mCherry^p^°^s^ colonies per square centimetre (Fig. 3B,D). Since the PAPA coatings used for peptide screening were modified with solutions of test peptides that were 100-fold more concentrated than the minimum threshold that had been determined for hPSC-supporting cRGDfK-PAPA coatings (1 μM, results not shown), the peptide screen was not repeated.

The only peptide-modified polymer coatings that appeared to support hPSC cultures in E8 medium were those modified with cRGDfK. Although cRGDfK is commonly considered to interact with αvβ3 integrins^1^, reports of the presence of αvβ3-integrins on hPSC cell surfaces are inconsistent and cRGDfK was optimised to mimic vitronectin, which also binds αvβ5-integrins^13,14^. In order to assess whether hPSC adhesion to cRGDfK-PAPA in the present study was mediated by integrins, the surfaces of hPSCs were stained for αvβ3- and αvβ5-integrins. The presence of αvβ3 and αvβ5 integrins were detected by flow cytometry on the surface of MDA-MB-435 control cells. In contrast, αvβ5 but not αvβ3 integrin was detected on the surface of H9 hPSCs (Supporting Information Figure S4).

Due to the low peptide concentrations required to produce cRGDfK-PAPA coatings that reliably allowed attachment and proliferation of hPSCs (data not shown), the associated cost-reduction, the reported preference of hPSCs for stiffer substrates^15^ and the ability to modify these surfaces with any azide-functionalised molecule, cRGDfK-PAPA-30UV coatings were selected for further experimentation and comparison with commercially available substrates.

### Characterisation of synthetic hPSC culture surfaces

**Figure 4:**
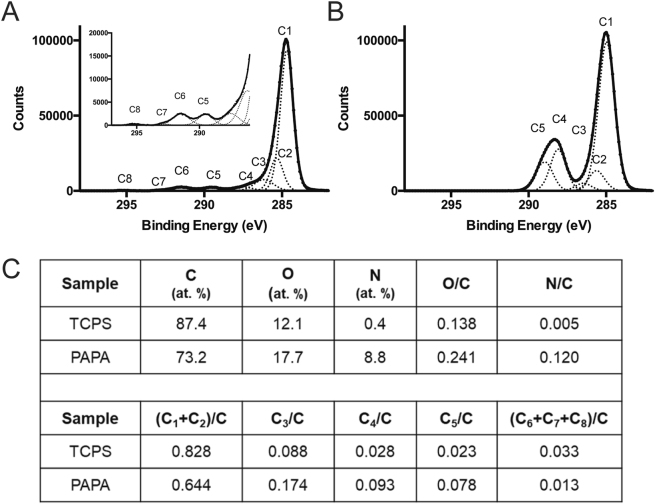
Characterisation of the chemical properties of PAPA coatings used for hPSC culture. (**A**) High resolution C 1 s XPS spectra for the TCPS surface and (**B**) a PAPA coated surface of 25 cm^2^ flasks. Here C_1_ and C_2_ refer to primary and secondary shifted C-C/C-H groups respectively, C_3_ refers to C-O/C-N groups, C_4_ refers to C=O/N-C=O groups, C_5_ refers to O-C=O groups and C_6_, C_7_ and C_8_ arise from the presence of aromatic carbon (very evident in TCPS). **(C)** XPS surface analysis results obtained for TCPS T25 surfaces and PAPA surfaces. The top half of the table contains quantitative elemental analysis results and elemental ratios whereas the lower half of the table contains the results from curve-fitting high-resolution C 1 s spectra (the C_x_ labels have the same significance as those in parts A and B containing example C 1 s spectra).

To verify the presence of coatings of the expected composition and chemistry, X-ray photoelectron spectroscopic (XPS) analysis was performed on the surface of modified culture flasks. The surface composition and high-resolution carbon 1 s spectra obtained from both uncoated and PAPA-30UV coated flasks are presented in Fig. 4. The atomic composition and high-resolution C 1 s spectra obtained from the analysis gave clear evidence of the presence of a PAPA coating on the surface of a TCPS substrate; significant increases were seen in the nitrogen and oxygen content of the surface compared to TCPS, which contained lower O atomic percentages and a very small N atomic percent. Furthermore, fitting of the high-resolution C 1 s spectra gave a component fit that was consistent with the presence of a polymer coating containing acrylamide species. Strongly indicative of acrylamide species is the presence of an intense C_4_ component at a binding energy of approximately 288 eV, compared to TCPS. The presence of an over-layer coating is confirmed by a reduction of the intensity of components (C_6_-C_8_) specific to the underlying TCPS.

Surface analysis of Geltrex™, Synthemax™ and StemAdhere™ coatings was also carried out (results not shown). Briefly, the analysed results obtained from Geltrex™ and StemAdhere™ coatings were consistent with the presence of protein coatings of varying thickness, while the Synthemax™ coating was consistent with the presence of an adsorbed layer of acrylate polymeric material with a thickness greater than the XPS sampling depth (>10 nm).

### Appearance and proliferation rates of hPSC cultures on synthetic surfaces

The potential of cRGDfK-PAPA coatings as hPSC culture surfaces was assessed using the H9, hiPS-NHF1.3 and Genea-02 hPSC lines. Starter cultures were thawed from banks of validated hPSC lines, which had been maintained for ten passages in E8 medium on Geltrex™-coated flasks to adapt cells to control conditions (Supporting Information Figure S5). Thawed cultures were passaged twice on Geltrex™ to allow recovery from the thawing process before being split into parallel cultures on each of the test surfaces.

**Figure 5:**
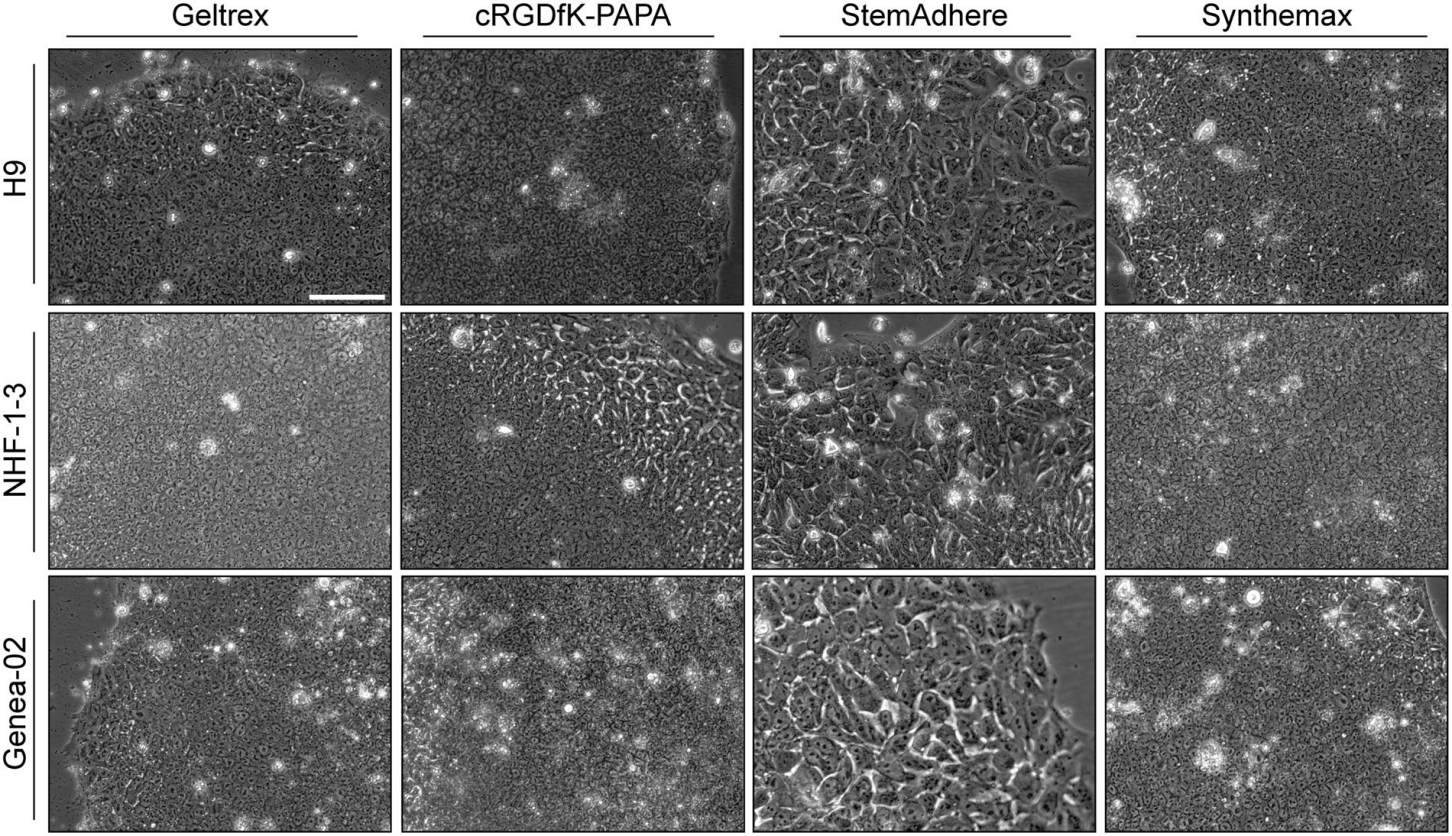
Morphology of hPSC cultures during long-term maintenance on defined culture surfaces. Phase contrast images of (top row) H9, (middle row) hiPS-NHF-1-3 and (bottom row) Genea-02 hPSCs cultured for ≥10 passages on (first column) Geltrex™, (second column) cRGDfK-PAPA, (third column) StemAdhere™ and (final column) Synthemax™. The cells maintained on StemAdhere™-coated surfaces appear to be flatter or larger than hPSCs on the other surfaces, which are morphologically indistinguishable from each other. The scale bar (A) represents 100 μm for all images.

Cell lines for experimentation were then maintained over ten passages in flasks coated with either cRGDfK-PAPA, StemAdhere™, Synthemax™ or Geltrex™, all hPSC cultures consistently formed tightly-packed colonies which displayed a typical range of hPSC morphologies (Fig. 5). Generally, flasks coated with cRGDfK-PAPA or Synthemax™ contained hPSCs that were morphologically indistinguishable from control cells maintained in Geltrex™-coated flasks. However, cultures maintained in StemAdhere™-coated flasks were observed to seed as single cells or smaller colonies than on the other surfaces and then to form colonies that appeared to be flatter and less tightly-packed (Fig. 5). These observations were consistent with observations made for H9-*OCT4*^*2AChryIM/w*^ cells maintained for 3 days on each surface (Supporting Information Figure S6).

**Figure 6:**
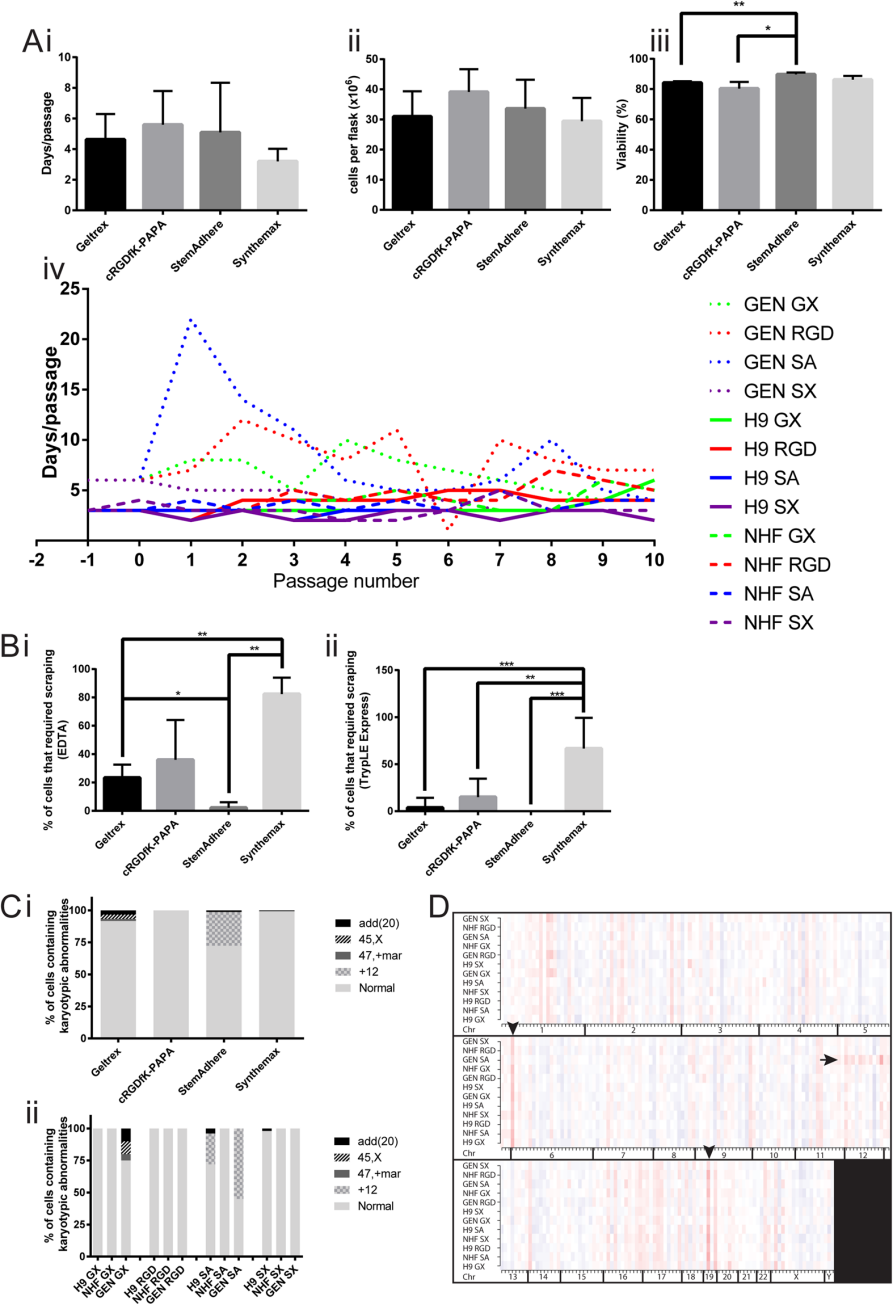
Growth dynamics and karyotypic stability of hPSC cultures maintained on different culture surfaces. (**A**) (i) The number of days taken for cultures of H9, hiPS-NHF1.3 (NHF) and Genea-02 (GEN) hPSCs to reach passaging density on surfaces coated with Geltrex™ (GX), cRGDfK-PAPA (RGD), StemAdhere™ (SA) or Synthemax™ (SX) is presented alongside (ii) the mean number of cells harvested at the end of each passage and the (iii) viability of harvested cells, all grouped by surface type. Mean +/− SD, n = 30 (10 passages for each of 3 cell lines). *p $\le$ 0.05, **p ≤ 0.01. (iv) The number of days taken for each passage to reach harvesting confluence is also presented for every passage of each cell line on each surface. (**B**) hPSC cultures were harvested with EDTA for continuing culture, and occasionally harvested with TrypLE™ Express for flow cytometric analysis. After treatment with a dissociation agent and pipetting with media, cells that remained adherent were physically removed with a cell scraper. The proportion of cells that required scraping was estimated by visual assessment at each harvest and is presented for cultures treated with (**A**) EDTA (n = 30 harvests) or with (**B**) TrypLE™ Express (n = 8 harvests for Geltrex™ and n = 6 harvests for each of cRGDfK-PAPA, StemAdhere™ and Synthemax™). Means are presented with scale bars representing standard deviations. *p $\le$ 0.05, **p ≤ 0.01, ***p ≤ 0.001 by unpaired two-tailed t-tests. (**C**) G-banding karyotyping assessment of hPSCs maintained on defined culture surfaces is summarised. The type and frequency of karyotypic abnormalities observed are plotted and arranged. Karyotypes are represented by the following colours: black: partial duplication of chromosome 20 [add(20)], black stripes on grey: loss of one sex chromosome (45, X), dark grey: includes additional unidentifiable chromosome (47, + mar), grey crosshatch on pale grey: trisomy of chromosome 12 (+12), light grey: normal karyotype (i) by surface type and (ii) by surface type and then by cell line. Full karyotypes are shown in Supplementary Figures 11 and 12 (**D**) The PluriTest™ assay was applied to passage 10 cultures and generated a transcriptional karyotype, which compared the expression levels of groups of genes in sample data sets to homologous genes in the pluripotent reference data sets. A heat map is formed displaying cytobands in which overall gene expression levels are higher (darker red indicates higher expression) or lower (blue, with darker blue indicating lower expression) in samples than is predicted by the pluripotency model. Horizontal red lines (arrow) indicate upregulation of genes in each cytoband of an entire chromosome (Chr); vertical red lines (arrowheads) indicate examples of cytobands that are upregulated in all samples compared to the reference data set.

Proliferation rates appeared to be more affected by cell-line variation than by surface type, with the passage duration of Genea-02 cultures being particularly variable between and within cultures maintained on different surfaces (Fig. 6Ai). When Genea-02 cells were initially seeded onto StemAdhere™-coated flasks, a typical mix of single cells and small colonies were observed to adhere to the surface. However, from day 4 many of these small colonies and single cells detached, reaching an estimated nadir around day 7 (Supporting Information Figure S7). As few as 10 colonies per flask were observed to survive at this point, yet the colonies continued to expand. This initial adaptation of Genea-02 hPSCs to culture on StemAdhere™ was repeated a further three times with consistent results (data not shown). When the Genea-02 culture on StemAdhere™ reached day 21 the colonies were very large, with dense centres surrounded by borders of morphologically normal hPSCs (Supporting Information Figure S7D,H,L), yet they were only estimated to cover 5% of the surface of the flask. At this point the cultures were harvested using EDTA (0.5 mM) and wholly transferred to a single StemAdhere™-coated flask, from which the culture recovered and ultimately survived for ten passages (Fig. 6A). No significant differences were observed between hPSC cultures maintained on different culture surfaces in the number of days required for hPSC cultures to recover from each passage or in the cellular yield (Fig. 6Aii). Subtle changes in passage duration were also mirrored by changes in yield, and all cultures were harvested with consistently high cell viability (Fig. 6Aii–iv), which further indicated that hPSC proliferation was consistent across all culture surfaces.

Cultures of hPSCs maintained on StemAdhere™-coated flasks were also observed to contain regions where the cells were highly compacted and colonies appeared to pile up on top of each other, as well as regions with well-defined borders where cells did not adhere (data not shown). Such phenomena were not observed in hPSC cultures maintained on other surfaces. While cultures maintained on StemAdhere™ lifted readily after shorter incubations in EDTA than were required to remove hPSCs from other surfaces, cell scraping was occasionally required to remove low numbers of cells from cRGDfK-PAPA and Geltrex™-coated surfaces during EDTA-mediated harvesting. However, hPSCs were more difficult to dissociate from Synthemax™, with cell scraping commonly required during harvesting with EDTA or TrypLE Express (Fig. 6B).

### Genetic stability of hPSCs maintained on synthetic culture surfaces

Genetic stability of all hPSC cultures was assessed by G-banding karyotyping (Fig. 6C,i–ii) and using the “transcriptional karyotype” feature of the PluriTest™ assay (Fig. 6D). The transcriptional karyotype is a heat map that presents gene expression levels arranged by genetic locus and illustrates whether the genes located within each cytoband are up- or down-regulated compared to the reference data set.

All hPSC cultures that had been maintained on cRGDfK-PAPA were observed to be karyotypically normal by G-banding karyotyping (Fig. 6C). However, trisomy of chromosome 12 was detected at a moderate frequency in Genea-02 (11/20 cells) and H9 (7/20 cells) cultures maintained on StemAdhere™. Trisomy-12 was also detected in the Genea-02 line by PluriTest™ (Fig. 6D, arrow), but the matching abnormality was not detected in the H9 population. This indicates that transcriptional karyotyping may be suitable for identifying highly abnormal populations of cells, but it may not be sensitive enough for early detection of low-frequency abnormalities. It was also interesting to observe genetic loci that were differentially regulated between cultures in this study and the reference data sets, probably resulting from the use of a different culture media to that used to originally populate the PluriTest™ dataset (Fig. 6D, arrowheads).

Additional low-frequency chromosomal abnormalities were only detected by G-banding (Fig. 6C,i–ii) and were predominantly amplifications or duplications of chromosomes 12 and 20, which have been commonly observed in a range of hPSC cultures^16^ and result in selective advantages^17^. Karotypically abnormal cells detected at low frequencies (as observed in the H9 culture on Synthemax™) indicated that the abnormalities either arose towards the end of the culture period or did not have a selective advantage. Chromosome spreads for all cultures are shown in Supplementary Figures 11 and 12.

### Maintenance of pluripotency of hPSC cultures on synthetic culture surfaces

**Figure 7:**
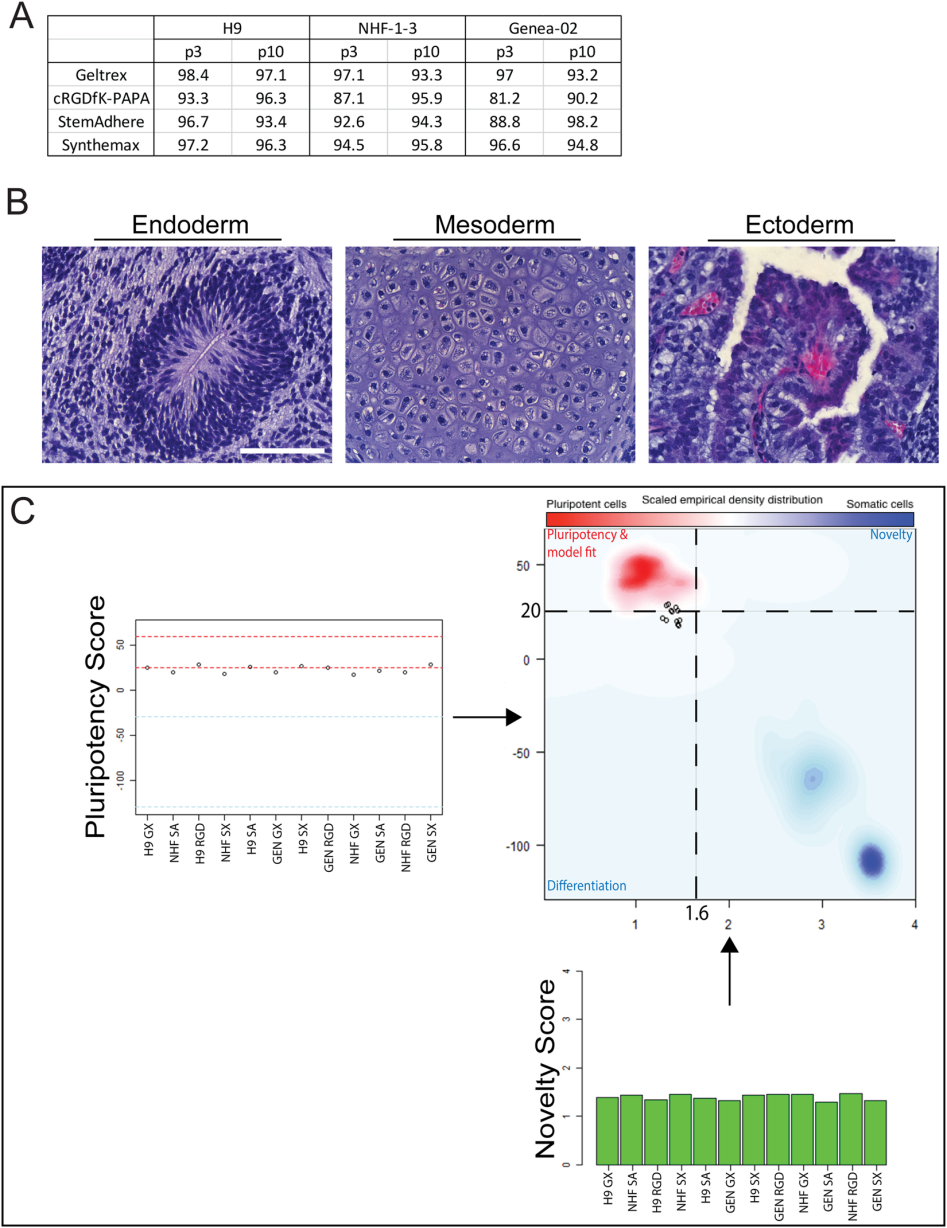
Maintenance of pluripotency of hPSC cultures on synthetic surfaces. H9, hiPS-NHF1.3 (NHF) and hES Genea-02 (GEN) hPSCs were maintained for ten passages on surfaces coated with Geltrex™ (GX), cRGDfK-PAPA (RGD), StemAdhere™ (SA) or Synthemax™ (SX). (**A**) Cells from each culture at passages 3 (p3) and 10 (p10) were immunostained for OCT4 and assessed by flow cytometry. This table presents the proportion of cells that were AF488-OCT4^pos^ relative to cells stained with appropriate isotype controls. (**B**) Representative images of ectodermal, endodermal and mesodermal structures that were identified in teratomas formed from the *in vivo* introduction of H9, hiPS-NHF1.3and Genea-02 cells following 10 passages on surfaces coated with Geltrex™, cRGDfK-PAPA, StemAdhere or Synthemax (full results presented in Supporting Information Figure S10). Scale bar = 100 μm. (**C**) The PluriTest™ assay produced Pluripotency Scores of these samples are plotted (top-left) against 95% confidence intervals for pluripotent (between the red lines) and non-pluripotent (between the blue line) populations. Novelty Scores are plotted (bottom right) and samples are colour-coded green (pluripotent), orange (uncertain) or red (not pluripotent) based on the probabilities given from the logistic regression model. Both scores are also represented together on a scatter plot (top-right). Here the samples (hollow circles) are compared to pluripotent reference data sets (red cloud, top-left quadrant) and differentiated and/or novel reference data sets (blue clouds, bottom-right quadrant).

Flow cytometric analysis at passage 3 & 10 for each cell line on each surface detected OCT4 in at least 80% of cells in each population compared to appropriate isotype controls (Fig. 7A, Supporting Information Figure S8). These findings were inconsistent with an earlier report that OCT4 protein was lost from hPSCs maintained on cRGDfK-presenting culture surfaces^10^ Furthermore, each hPSC culture passed teratoma assays for the *in vivo* formation of tissues representing the three embryonic germ layers (Fig. 6B, Supporting Information Figure S9) and the PluriTest™ pluripotency assay; all samples produced Novelty Scores and Pluripotency Scores typical of hPSC populations (Fig. 7C). Minor variations between the Pluritest™ data generated from this experiment and the reference data could be attributed to a recently observed technical drift in the hybridisation chemistry of the Illumina HT 12V4 microarray platform, as well as an offset due to a new microarray scanner generation (iScan) that was used for these samples (Franz-Josef Müller, Zentrum für Integrative Psychiatrie Kiel, Germany, *pers*. *comm*.). The PluriTest™ assay also generated a phylogenetic tree, which indicated that most hPSC lines maintained on cRGDfK-PAPA were more closely related to control culture maintained on Geltrex™ (Supporting Information Figure S10) than to other cell lines.

## Discussion

The results presented herein show that our method was capable of generating surface coatings that were well-defined in terms of their composition, and to which peptides could be readily covalently attached via at least two well-understood coupling chemistries [water soluble carbodiimide and Cu(I)-mediated click coupling]. The coatings were stable, could be sterilised using industry standard methodologies, and were readily applicable to maintaining hPSC cultures. E8 culture media incubated for 66 h in PAAA- and PAPA- coated wells did not display cytotoxic properties when applied to fresh hPSC cultures (data not shown). Also, following appropriate peptide modification, the coatings readily supported both attachment and maintenance of hPSCs cultures.

It was found that hPSCs bound more efficiently to cRGDfK-modified PAAA and PAPA coatings than they did to coatings modified with other test peptides. A culture system comprising E8 medium and cRGDfK-PAPA coatings was then shown to support the long-term maintenance of undifferentiated hPSC cultures similar to control cultures on Geltrex™. hPSC cultures were karyotypically stable over ten passages when maintained on cRGDfK-PAPA-coated surfaces, while cultures on Geltrex™, StemAdhere™ and Synthemax™ all displayed karyotipic abnormalities to varying degrees. These findings will contribute to the ongoing development of peptide-presenting surfaces for the maintenance of hPSC cultures.

Of the 37 peptide-presenting polymer coatings assessed, only cRGDfK-modified PAAA and PAPA coatings appeared to be feasible for use in hPSC culture. This finding was surprising, since maintenance of hPSC cultures has not previously been reported on surfaces that present cRGDfK-alone, despite the widespread use of cRGDfK in various applications including *in vitro* adhesion of osteoblasts^18^, chondrocytes^19,20^ and melanoma cells^14^. Only one other publication had reported an attempt to maintain hPSC cultures on such a surface^10^.

The culture surface produced by Klim *et al*.^10^ presented biotinylated cRGDfK peptides from streptavidin-coated tissue culture-treated polystyrene. Although two hESC lines were reported to adhere to and proliferate on this surface for 3 passages, abnormal morphology, substantial morphological differentiation and loss of OCT4 were observed. On the other hand, the cRGDfK-modified coatings reported herein were consistently observed to support adhesion of large numbers of both hESC and hiPSC colonies and were able to maintain karyotypically normal OCT4-positive hPSC cultures for at least ten passages.

It was interesting to note that, in this study, the only peptide-modified polymer coatings that appeared to be feasible for use as hPSC-supportive culture surfaces were those modified with cRGDfk. This finding differs from a report by Klim *et al*.^10^, who reported that cRGDfk was not a reliable hPSC culture surface. A possible reason for the poor performance of the cRGDfK-presenting surface reported by Klim *et al*.^10^ is instability of the underlying substrate. Although biotin-streptavidin interactions are some of the strongest non-covalent bonds (bond-dissociation energy of −18 kcal/mol), carbon-carbon covalent bonds are much stronger (−83 kcal/mol)^21^. αv-containing integrins have been observed to selectively localise with high force regions within focal adhesions when bound to linear RGD^22^, and to break biotin-avidin bonds when bound to cRGDfK^23^. Although no loss of cellular attachment was observed in the study by Jurchenko *et al*.^23^, the experiments were performed over a shorter time frame and likely with a lower starting ligand density. It is therefore conceivable that integrin-mediated removal of peptide ligands could have affected cell adhesion in the study by Klim *et al*., while the more diffuse HSPGs may have exerted less force and not damaged the surface^10^.

The requirement of Y-27632 supplementation for hPSC culture on Klim’s cRGDfK-presenting surface may reflect the role of Y-27632 in inhibiting myosin contractility^10,24^, which would inhibit cell-mediated destruction of the surface. Conversely, PAAA and PAPA are covalently bonded from the peptide ligand through to the underlying polystyrene and so physical removal of cRGDfK peptide ligands by cellular forces in the current study is highly unlikely.

When coatings were modified with three of the remaining four RGD-containing ECMP-derived test peptides (11, osteopontin; 34, bone sialoprotein; 35, vitronectin), low numbers of hPSCs were observed to bind (Supporting Information Figures S4 and S5). Poor adhesion of hPSCs to pep34-PAAA and pep35-PAAA coatings was particularly surprising, since these peptides have been presented on surfaces previously reported to support hPSC culture, including Corning Synthemax™^25,26^.

During the development of Corning Synthemax™, acrylate coatings were modified with peptides *via* a carbodiimide chemistry and using peptide concentrations similar to those used to modify PAAA coatings in the present study^26^. A reduction in hPSC adhesion efficiency to peptide acrylate coatings was observed when the surfaces were modified with solutions containing concentrations of peptide between 250 and 500 μM in the Melkoumian study^26^. However, increasing the peptide concentration used to modify pep34-PAAA and pep35-PAAA surfaces to 600 μM did not result in levels of hPSC adhesion equivalent to cRGDfK-PAAA surfaces that had been modified with solutions containing only 200 μM of peptide (Supporting Information Figure S2A). Another recent study reported similar input peptide requirements and stated that it was “necessary to use high concentration of oligoVN [peptide 34]” to mediate hPSC adhesion^27^.

Furthermore, a recent study found that culture media spiked with cyclic RGDfC and RGDfV peptides blocked hPSC adhesion more effectively than media spiked with linear RGD peptide^28^. Peptide-cell interactions can be affected by ligand-CAM affinity and/or integrin subtype specificity. The RGD motif has been reported to bind eight of the 24 known integrin subtypes, including αvβ3, αvβ5 and α5β1^29,30^. The CAM-ligand affinity and integrin subtype specificity of the RGD motif are conformation-dependent and can be affected *in vivo* by flanking sequences, tertiary structure and post-translational modifications, although the latter two are missing from the short, linear, synthetic peptides used in this study^31^. It is therefore somewhat unsurprising that surfaces modified with the synthetically-derived cRGDfK, for which integrin affinity has been optimised *in vitro* in the absence of post-translational modifications or a surrounding protein structure, bound hPSCs more efficiently than RGD-containing peptides that have been isolated from the *in vivo* environment. Essentially, the cyclic structure of the cRGDfK peptide mimics the tertiary structure in the native vitronectin protein as well as providing chemical stability.

It has previously been reported that cyclic peptides containing the RGDf sequence specifically interact with αvβ3 integrins^1^, that undifferentiated hPSC cultures attach to but are unable to be maintained on cRGDfK-presenting surfaces^10^ and that hPSCs are unable to bind to surfaces that only present αvβ3-specific ligands, but can adhere to vitronectin-coated surfaces (αvβ3- and αvβ5-binding)^14^. Unfortunately cRGDfK-modified surfaces were not included in the latter study. However, reports of hPSC integrin expression profiles are inconsistent, and the specificity of cRGDfK-αvβ3 integrin interactions has not been demonstrated directly^32–34^. The inability to detect αvβ3 integrin on undifferentiated hPSCs by flow cytometry (Supporting Information Figure S4) was consistent with the report by Klim *et al*.^14^, but not with results from an earlier study by Meng *et al*.^13^. Subsequent studies using antibody-blocking experiments demonstrated that hPSC interactions with the Synthemax™ culture surface are predominantly mediated by αvβ5 integrins, not αvβ3^28,35^ and it has also been reported that blocking αvβ3 integrins had no observable effect on hPSC adhesion to Matrigel™^36^. Our findings that αvβ5 integrin is detected, and αvβ3 integrin is not, by flow cytometry on the surface of H9 hESCs are entirely consistent with these results (Supporting Information Figure S4). These results collectively indicate that hPSC adhesion to cRGDfK-presenting polymer substrates is likely to be mediated by αvβ5 integrins rather than αvβ3 integrins.

Regardless, hPSC adhesion efficiency to cRGDfK-modified PAAA and PAPA-coated surfaces was greater than surfaces modified with other test peptides. These results indicate that cRGDfK is an ideal peptide for mediating hPSC adhesion to peptide-presenting polymer coatings.

Eleven polymer coatings were modified with HSPG-binding peptides. Of these, hPSCs were only observed to inefficiently and unstably bind to pep20-PAPA and pep31-PAPA. Such a poor result was particularly interesting for pep31-PAPA, since peptide 31 is a vitronectin-derived peptide that has been included in a range of hPSC culture surfaces^10–12,37^. Nevertheless, hPSC adhesion to pep31-PAPA remained low despite attempts to optimise reaction conditions (Fig. 3).

The observation that hPSCs bound more efficiently to surfaces modified with RGD-containing peptides (34, 35 and cRGDfK) than to surfaces modified with HSPG-binding peptides is inconsistent with a previous report of hPSC adhesion to self-assembled monolayer (SAM) of alkanethiols that had been modified at a high density with any of 9 different peptides^10^. It was observed that a lower density of peptide 31 was required to mediate hPSC adhesion to the SAMs than of other (integrin- or HSPG-binding) peptides. This “low” peptide density was still 1000-fold higher than the threshold surface peptide density for hPSC attachment to cRGDfK-PAPA (data not shown). It is interesting to note that every report of hPSC culture on surfaces that present peptide 31 or other HSPG-binding peptides has used culture medium supplemented with Y-27632 or other Rho pathway inhibitors^10–12,15,38–40^.

The downstream effects of Y-27632 are unclear^41^ and may include effects on cell fate bias^42^ and increasing cell-cell interactions and adherence^43^. Surfaces presenting a combination of HSPG- and integrin-binding ligands have been reported to obviate the requirement for Y-27632 supplementation^10,13^. However the present study is the first in which surfaces modified with cRGDfK-alone have been reported to mediate adhesion of hPSCs in the absence of Y-27632 supplementation, let alone outperform coatings modified with HSPG-binding peptides. The formation of multilayered aggregates, detachment and loss of OCT4 observed in hPSCs cultured on pep31-PAPA (Supporting Information Figure S3) resembled observations made about hPSCs binding to the cRGDfK-biotin-streptavidin surface developed by Klim *et al*.^10^ So Y-27632 supplementation may compensate for inadequate hPSC culture surfaces in a ligand- and receptor-independent fashion.

Other peptides that have previously been reported to feature in hPSC-binding surfaces but failed to do so in the current study included randomly generated peptides (4 and 5) that were identified from a phage display library^40^. They may have been affected by the use of conditioned medium generated from feeder culture of mitotically inactivated mouse embryonic fibroblast and/or by cross-contamination between adjacent array elements^44^. Conducting the current screen in individual wells instead of on a micro-patterned surface reduced the risk of such contamination. Surfaces modified with laminin-derived peptides (16, 17 and 30) were only observed to mediate hPSC adhesion when applied in combination with BSA-treated surfaces^13^. This is the first follow-up publication using surfaces modified with these peptides and the results support the hypothesis that laminin-derived peptides are individually inadequate for supporting maintenance of undifferentiated hPSC cultures.

Since hPSCs bound more efficiently to surfaces modified with cRGDfK than those modified with any other peptide, a long-term study was conducted using cRGDfK-PAPA. Overall, hPSC cultures that had been maintained for at least ten passages on the cRGDfK-PAPA coating were comparable to cultures maintained in parallel on the commercially available culture surfaces for the presence of OCT4 (Fig. 7A), gene expression (Fig. 7C) or *in vivo* differentiation potential (Fig. 7B).

Aside from the quality of the cell product, factors worth considering when selecting a scalable surface for maintaining hPSC cultures include cost, preparation, shelf life and logistical challenges involved in passaging cells (e.g. cell scraping). The four test surfaces have been ranked for these parameters in Table 1 below. Although our coating approach is readily scalable in theory, cRGDfK-PAPA-coated flasks have only actually been produced at relatively small scale, so the commercial cost of cRGDfK-PAPA could not be calculated.

**Table 1:** Geltrex™, cRGDfK-PAPA, StemAdhere™ and Synthemax™ are ranked as hPSC culture surfaces from most (1) to least (4) preferred with regards to cost, preparation, shelf life and the requirement for scraping to harvest cells. For example, Geltrex is the cheapest and is therefore ranked 1 (most preferred) for cost, while StemAdhere^TM^ requires the least scraping and is therefore ranked 1 for scraping. Cost was ranked according to a recently published comparison^57^, preparation was ranked according to the number of steps involved in the coating procedures at the point of passage (commercially available cRGDfK-PAPA would be prepared as a complete coating onto which cells would be seeded directly), the reported shelf lives of commercially available surfaces were compared to the tested shelf life of cRGDfK-PAPA (data not shown) while the cell scraping rankings are a summary of the results presented in Fig. 6B.

Surface	Cost	Preparation	Shelf life	Cell scraping
Geltrex™	1	4	3	2
cRGDfK-PAPA	Unknown (likely less than Synthemax™)	1	1	2
StemAdhere™	2	3	4	1
Synthemax™	3	2	2	4

Nevertheless, efficient hPSC adhesion has been consistently observed on cRGDfK-PAPA coatings modified with concentrations of cRGDfK as low as 1 μM (data not shown), which reflects a potential saving of peptide cost as great as 500-fold and indicates that cRGDfK-PAPA is likely to be less expensive to produce than Synthemax™^26^.

The commercial benefits of low-cost surfaces that require little preparation and have a long shelf life are self-evident. Although cell scraping is included in some hPSC passaging protocols^45–47^, such manual harvesting is not easily scalable and could not be applied to multilayer cell stackers, cell factories, or three-dimensional cell culture systems including microcarriers and hydrogels^48^.

Aside from the logistical issues involved in large scale hPSC cultures, the use of cell scraping is also undesirable because the surface coating is likely to be harvested along with the cells, particularly for non-covalently bonded products such as StemAdhere™ and Synthemax™. The implications for incorporation of any coating-derived contaminants would therefore need to be assessed prior to the application of cells cultured in these systems to cell-based diagnostic assays and therapies. In the current study, hPSCs maintained in cRGDfK-PAPA-coated or Geltrex™-coated flasks were observed to require harvest by cell scraping significantly less often than cultures on Synthemax™-coated flasks, while hPSCs maintained in StemAdhere™-coated flasks lifted readily after a shorter incubation in EDTA. The ease of harvesting hPSCs from StemAdhere™-coated flasks, the associated improvement in viability (Fig. 6A), and the short shelf life compared to other coatings (Table 1) may reflect a reduced coating stability.

## Conclusions

PAAA and PAPA coatings were modified with 36 peptides which had previously reported roles in cell adhesion and then screened for adhesion of hPSCs. HPSCs were observed to bind more effectively to cRGDfK-modified surfaces than surfaces modified with any of 36 other peptides with previously reported roles in cell adhesion. A greater number of hPSCs were consistently observed to adhere to cRGDfK-PAAA and cRGDfK-PAPA coatings than to coatings modified with any other peptide, including those used in the commercially available hPSC culture surface Synthemax™. HPSCs did not attach to surfaces modified with HSPG-binding peptides, which may have been due to the absence of ROCK inhibition. The chemically defined and relatively affordable cRGDfK-PAPA coating has been demonstrated to maintain high quality hPSC cultures over long-term culture. Cultures of three hPSC lines that were maintained in cRGDfK-PAPA-coated flasks remained comparable to control cultures maintained in parallel in Geltrex™-coated flasks in terms of growth rate and maintenance of pluripotency as assessed by cell morphology, gene expression and teratoma formation. Cultures maintained in parallel on the commercially available chemically defined surfaces StemAdhere™ and Synthemax™ were also observed to remain pluripotent by these measures. Detailed analysis of proliferation rates was obscured by cell line variation, particularly in the Genea-02 cell line. Importantly, hPSC cultures maintained on cRGDfK-coated surfaces demonstrated less karyotypic abnormality than cultures on the other surfaces tested, although more detailed analysis would be required to confirm this observation. Trisomy of chromosome 12 arose frequently and only in hPSC cultures maintained on StemAdhere™-coated surfaces, indicating that this surface may predispose hPSC cultures to *in vitro* selection pressures and possible genetic instability. Cultures maintained on Synthemax™ in this study would not have been suitable for large scale production due to the need for physical harvesting of cells by cell scraping, although this may be ameliorated in future by novel dissociation reagents.

The results of this study collectively indicate that cRGDfK-PAPA has the potential to provide a viable, commercially scalable surface for the support of human pluripotent stem cells.

## Experimental Section

### Cell culture

#### hPSCs

The hESC-WA09 (H9)^49^, hESC-GENEA-02^50^, and hiPS-NHF1.3^51^ cell lines were respectively provided under materials transfer agreements by the Wisconsin Alumni Research Foundation (WARF) and Genea, and kindly by Prof. Ernst Wolvetang (Australian Institute for Bioengineering & Nanotechnology). All hPSCs were maintained in Essential 8™ (E8) medium (Life Technologies) on Geltrex™-coated tissue culture polystyrene at 37 °C in an atmosphere containing 5% CO_2_ and passaged using EDTA. Cells that did not lift from pipetting with medium after incubation in EDTA (0.5 mM) were manually removed from the surface using a cell scraper. HPSC cultures for these experiments were thawed from banks of cryopreserved H9, hiPS-NHF1.3 and Genea-02 hPSCs that had been adapted to culture in E8 medium on Geltrex™-coated surfaces for at least ten passages. All work using hPSCs was carried out in accordance with approvals from Monash University (Project ID 2963) and the CSIRO Human Research Ethics Offices.

#### MDA-MB-435 melanocytes

The melanocyte cell line MDA-MB-435 was kindly provided by A/Prof John Price (Victoria University, Melbourne, Australia). MDA-MB-435 cells were maintained in high glucose DMEM supplemented with 10 v/v % FBS and passaged using TrypLE™ Express.

#### Generation of H9-*OCT4*^*2AChryIM/w*^ reporter cell line

An *OCT4*^*2AChryIM/w*^ targeting vector was constructed by amplifying 5.4 kb (left homology arm) and 3.5 kb (right homology arm) of genomic DNA 5′ and 3′ of the OCT4 stop codon respectively. These fragments were cloned sequentially into pCR™-Blunt II-TOPO™ (*Life Technologies*) such that each arm was separated by a single *AscI* restriction site. Conventional cloning was then used to insert a cassette comprising sequences encoding a T2A peptide fused to mCherry followed by an internal ribosomal entry site located upstream of a synthetic neomycin resistance (*NeoR*) gene - a version of *NeoR* that had been optimised for expression in mammalian cells (designated Meo). The structure of the final vector is shown in Fig. 1. To enhance the targeting frequency for this vector, plasmids encoding TALENs directed against sequences immediately 3′ of the OCT4 stop codon (Cellectis) were co-electroporated with linearised OCT4-mCherry vector into H9 hESCs using previously published protocols^52^. G418 resistant colonies were isolated as described previously^52^ and screened for gene targeting events using a PCR based approach that utilised a forward primer directed against sequences within *Meo* in conjunction with a primer homologous to GAPDH genomic sequences downstream of the 3′ most sequences contained within the targeting vector. PCR of correctly targeted clones yielded a PCR fragment of 3.6 kb. Five of six mCherry^pos^ colonies screened were positive for the diagnostic PCR product.

### Synthesis of PAAA and PAPA coatings on TCPS

Acrylic acid was purified by short-path distillation to remove dimers and inhibitors, and then stored at −20 °C until use. Propargyl acrylamide was prepared according to a published method^53^. Monomer solutions and polymer coatings were prepared in an oxygen-free environment. To prepare PAAA coatings, co-monomer solutions containing 7.5% w/v acrylamide (60 mole %) and acrylic acid (40 mole %) were prepared in MQ H_2_O. To prepare PAPA coatings, co-monomer solutions containing 10% w/v acrylamide (70 mole %) and propargyl acrylamide (30 mole %) were prepared in MQ H_2_O. Tissue culture-treated polystyrene vessels (either multi-well or tissue culture flasks) were coated with 150 μl/cm^3^ of the appropriate mixed monomer solutions and transferred to bags that were subsequently vacuum-sealed. Surface initiated polymerisation from the TCPS substrate materials, covered by monomer solutions and sealed inside a low-oxygen environment, was induced with repeated exposure to high power UV light generated by dual Light Hammer® 6 UV lamps (Fusion UV) at set at 70% power. Following UV treatment, polymer-coated plates were removed from the vacuum-sealed bags and washed immediately and vigorously in MQ H_2_O until visible monomer and polymer solution were removed. Plates were then soaked for three days in a large volume of MQ H_2_O, which was changed daily. On the third day, polymer-coated plates were dried overnight in a laminar flow hood. Dry plates were sterilised with gamma irradiation at a dose of 15 kGy (Steritech, Dandenong, Australia) or by treatment with antibiotic-antimycotic solution (Life Technologies, Cat No: 15240) diluted 1:50 in DPBS-.

### Peptide screening approach

Unless stated otherwise, the following methods were used to prepare peptide-modified polymer coatings in 24-well plates for peptide screening.

#### Peptide modification of PAAA wells for peptide screening

The carboxylic acid functional groups present in PAAA coated plates, prepared using methods described above, were activated with an aqueous solution containing 125 mM 1-ethyl-3-(3-dimethylaminopropyl)carbodiimide (EDC) and 125 mM *N*-hydroxysuccinimide NHS, incubated overnight in a 200 μM solution of test peptide in DPBS- and washed thoroughly in DPBS-.

#### Peptide modification of PAPA wells for peptide screening

A surface-based CuAAC peptide conjugation method was developed based on previous work^54^. All reactions were performed at room temperature in aqueous solutions prepared with MQ H_2_O unless otherwise stated. CuAAC reaction solutions were prepared under atmospheric conditions and contained 100 μM copper sulphate, 272 μM sodium ascorbate and 200 μM azide-conjugated peptide in 1 M HEPES buffer unless otherwise stated. Polymer-coated surfaces were exposed to 400 µL/well of CuAAC reaction solution. Plates were sealed in airtight plastic pockets and incubated at room temperature for 24 hours while shaking at 100 rpm. Peptide-coupled wells were washed thoroughly in DPBS-, treated with 0.1 M Na_2_EDTA overnight and again thoroughly washed in DPBS-. Peptide-modified surfaces were typically stored for no more than three months at 4 °C under DPBS- containing 50 U/mL penicillin/streptomycin and wrapped in parafilm.

Peptides were produced by Genscript Inc. (Piscataway, NJ), Peptides International Inc. (Louisville, KY), Mimotopes Pty (Notting Hill, Australia), or at CSIRO by solid-state synthesis.

Briefly, all peptides were synthesised manually on fluorenylmethyloxycarbonyl (Fmoc)-Ala-Wang (loading: 0.72 mmol/g), Fmoc-Arg(Pbf)-Wang (loading: 0.38 mmol/g), Fmoc-Gln(Trt)-Wang (loading: 0.61 mmol/g) or Fmoc-Gly-Wang (loading: 0.8 mmol/g) resin at 0.1 mmol scale. For manual synthesis operations, the peptide resin was placed in a syringe reactor fitted with a porous polyethylene frit (Sigma Aldrich). The resin was pre-swollen in N,N-dimethylformamide (DMF; Merck) for 30 minutes and then filtered. The following Fmoc-deprotection and coupling steps were carried out until the desired sequences were synthesised.

*Fmoc-deprotection:* a mixture of piperidine (Sigma Aldrich) and DMF (20:80, v/v) was added to the resin. The resin was shaken for 5 minutes and filtered and the fresh portion of the mixture of piperidine and DMF (20:80, v/v) was added to the resin. The resin was shaken for 15 minutes, filtered and washed with DMF (3 × 5 minutes), Dichloromethane (DCM; Sigma Aldrich, 3 × 5 minutes) and DMF (3 × 5 minutes).

*Coupling step:* Three-fold excesses of Fmoc-L-amino acids; 2-(1H-benzotriazole-1-yl)-1,1,3,3-tetramethyluronium hexafluorophosphate (HBTU) and N-hydroxybenzotriazole (HOBt; Sigma Aldrich), in the presence of double the molar amount relative to Fmoc-L-amino acid of N,N-diisopropylethylamine (DIEA; Sigma Aldrich), were used for the coupling steps, with DMF as solvent. The resin was shaken for 1 hour at RT. The resin was then filtered and washed with DMF (3 × 5 minutes), DCM (3 × 5 minutes) and DMF (3 × 5 minutes).

*Cleavage protocol:* Peptide resin was treated with 5 mL of cleavage mixture: Trifluoroacetic acid (TFA; Sigma Aldrich)/ Triisopropylsilane (TIS; Sigma Aldrich)/H_2_O (95/2.5/2.5, v/v/v); 90 minutes, RT. The cleavage solution was separated from the resin by filtration and peptides were precipitated by addition of chilled diethyl ether (Sigma Aldrich), centrifuged, and decanted. The product peptides were washed twice with the same solvent. Samples were then left under vacuum for 30 minutes. Peptides were dissolved in MQ H_2_O and lyophilised.

*Analysis and Purification:* Analytical reversed-phase high performance liquid chromatography (HPLC) was performed as described above. Preparative HPLC was performed on C18 column (21.2 × 250 mm, 10 μm, Phenomenex, Torrance, CA) in a model Agilent 1260 Infinity system (Agilent Technologies, Santa Clara, CA). Solvents A and B were 0.1% TFA (v/v) in MQ H_2_O and acetonitrile, respectively, and elution was with 5–70% linear gradients of solvent B into A over 25 minutes, at 15 mL/minute flow rate, with UV detection at 210 nm. Preparative fractions of satisfactory purity ( ≥ 95%) by analytical HPLC were pooled and lyophilised. All peptides were characterised for identity by HPLC analysis and mass spectrometry.

Polymer-coated 24-well plates were modified with test peptides, the lead cRGDfK peptide or (for PAAA plates only, the non-binding control cRADfK peptide. H9-*OCT4*^*2AChryIM/w*^ cells harvested from Geltrex™-coated maintenance flasks using EDTA were seeded in all polymer-coated wells at a density of 15 000 cells/cm^2^ in 400 µl of E8 media. Geltrex™-coated control wells seeded at equal and one-third density were included on a separate 24-well plate. Forty-eight hours after cell seeding, cultures were visually assessed under phase contrast microscopy and adherent colonies were counted. Daily media changes were gently performed from day 2 and cultures were observed daily. Colony growth was roughly assessed four days after cell seeding by re-scanning wells. Samples and controls were included in triplicate wells in randomised locations on each plate. Experiments were repeated three times using polymer coatings synthesised and modified independently and seeded with hPSCs harvested from different cultures. Peptides were considered to have failed the screen if no hPSCs were observed to bind to any of the wells modified with a peptide in either of the first two experiments. Colony counts from surfaces modified with each peptide were compared to counts from the inbuilt cRGDfK-modified controls.

### Preparation of hPSC culture surfaces for long term maintenance

A surface-based CuAAC peptide conjugation method was developed based on previous work^54^. All reactions were performed at room temperature in aqueous solutions prepared with MQ H_2_O unless otherwise stated. CuAAC reaction solution was prepared in atmospheric conditions and typically comprised 100 μM copper sulphate pentahydrate, 272 μM sodium ascorbate and 36 μM azide-conjugated cRGDfK peptide in 1 M HEPES buffer. Each PAPA-coated flask was treated with 4 mL of CuAAC reaction solution, sealed with plug seal caps and the reaction occurred over 24 hours while rocking at 20 rpm. cRGDfK-PAPA flasks were washed thoroughly in DPBS-, treated with 0.1 M Na_2_EDTA overnight, thoroughly washed again in DPBS, re-sealed and stored for no more than three months at 4 °C under DPBS- containing 50 U/mL penicillin/streptomycin.

StemAdhere™ and Synthemax™-coated flasks were prepared and stored according to manufacturer’s instructions.

### Flow cytometry

Extracellular and intracellular immunolabelling reactions and FACS analyses were performed as described previously^55^. Briefly, cell cultures were washed twice in DPBS- and then dissociated with TrypLE™ Express. For detection of αvβ3 and αvβ5 integrins, live, single cells were blocked in 10% goat serum for 30 min, followed by immunostaining for 1 hour on ice with anti-αvβ3 integrin antibody (clone VNR-1, abcam: ab78289) and anti-αvβ5 integrin antibody (clone P1F6, abcam: ab177004) diluted 20 µg/mL in FACS buffer or with an appropriate isotype control (Becton Dickinson, Cat No: 557273). For detection of intracellular OCT4, cells were first fixed in paraformaldehyde (Polysciences) then permeabilised in 0.1% Triton X-100 (Sigma Aldrich) and stained with anti-OCT4 antibody (Santa Cruz Biotechnology, Cat No: C-10) or with an appropriate isotype control (Becton Dickinson, Cat No: 559530). Immunostained cells were washed in FACS buffer, incubated for 20 minutes on ice with an appropriate secondary antibody (Alexa Fluor 488-conjugated goat anti-mouse IgG1 Life Technologies, Cat No: A-21121), washed again and analysed. Data were recorded on a Guava easyCyte™ 8HT sampling flow cytometer (Millipore), an LSR II (Becton Dickinson) or a FACSCalibur™ (Becton Dickinson) flow cytometer. Post-analysis was performed using FlowJo software. Spectral compensation for auto and non-specific fluorescence to determine fluorophore positive and negative cell populations was performed as previously described^55^.

### Microarray analysis of total RNA and PluriTest™

Lysate of undifferentiated hPSCs stored in Buffer RLT was thawed and the RNA was isolated using RNeasy Mini Kits (Qiagen) according to the Qiagen handbook entitled “RNeasy Mini Handbook 04/2006”. The quality and quantity of RNA was assessed against threshold OD_260_/OD_280_ ratios of greater than 1.8 and RNA Integrity Numbers (RIN) greater than 8. Microarray analysis was performed using HumanHT-12 v4 Expression BeadChip Microarrays (Illumina, San Diego, California) at the Australian Genome Research Facility (Parkville, Australia). (*.idat) were uploaded to www.pluritest.org for post-analysis.

### Karyotyping

G-banding karyotype analysis was performed by Southern Cross Pathology in the Cytogenetics department of Monash Medical Centre (Clayton, Australia).

### Teratoma assay

Teratomas were generated as previously described^56^. For each experiment, a cell/Matrigel™ suspension was injected into both testes of three mice. Tumours were histologically scored for the presence of tissue types representing endoderm, mesoderm and ectoderm. Test populations were deemed pluripotent if the three germ layers were collectively represented in any tumour or tumours derived from that population. Protocols and use of animals in this project were undertaken with approval of the Monash University Animal Welfare Committee following the 2004 Australian Code of Practice for the Care and Use of Animals for Scientific Purposes and the Victorian Prevention of Cruelty to Animals Act and Regulations legislation.

## Electronic supplementary material

Supporting Information
