# Supplementary material for: Long-Term Maintenance of Human Pluripotent Stem Cells on cRGDfK-Presenting Synthetic Surfaces
Source: Sci Rep. 2018 Jan 15;8:701. doi: 10.1038/s41598-018-19209-0 (PMC5768753; doi:10.1038/s41598-018-19209-0)
Supplement: Supplementary file 1 — Supporting Information [file 41598_2018_19209_MOESM1_ESM.doc]

# Supporting Information for:

# Long-Term Maintenance of Human Pluripotent Stem Cells on cRGDfK-Presenting Synthetic Surfaces

Jack W. Lambshead^1,2^, Laurence Meagher^3^, Jacob Goodwin^1,2^, Tanya Labonne^4^, Elizabeth Ng^4^, Andrew Elefanty^4^, Edouard Stanley^4^, Carmel M. O’Brien^1,2^, Andrew L. Laslett^1,2^*

1. CSIRO Manufacturing, Clayton, Victoria 3168, Australia.
2. Australian Regenerative Medicine Institute, Monash University, Victoria 3800, Australia.
3. Monash Institute of Medical Engineering, Monash University, Victoria 3800, Australia.
4. Murdoch Children’s Research Institute, The Royal Children’s Hospital, Victoria 3052, Australia.

* Correspondence: [andrew.laslett@csiro.au](mailto:andrew.laslett@csiro.au) (ALL)

# Supporting Information Table S1: The sequence and biological origin of each peptide is listed along with the polymer coating they were screened on, their biological origin and the cell receptors with which they’ve been reported to interact. Ac refers to acetylation of the N-terminal amine, kDa; kilodaltons, K(N3); azide-conjugated lysine, PAAA; poly(acrylamide*-co-*acrylic acid), PAPA; poly(acrylamide*-co-*propargyl acrylamide). Additional details can be found in Supporting Information Table S2.

| **#** | **Sequence** | **Surface** | **Peptide origin** | **Cell receptor** |
| --- | --- | --- | --- | --- |
| **1** | K(N3)GTTVKYIFR | PAPA | Laminin γ1-chain | Unknown |
| **2** | K(N3)GRNIAEIIKDI | PAPA | Murine laminin β2 chain | Unknown |
| **3** | Ac-KGRYVVLPR | PAAA | Laminin β1 chain | Glycosaminoglycan |
| **4** | K(N3)LTTAPKLPKVTR | PAPA | Phage display library | Unknown |
| **5** | K(N3)TVKHRPDALHPQ | PAPA | Phage display library | Unknown |
| **6** | Ac-KPHSRN | PAAA | Fibronectin | Integrin α_5_β_1_ |
| **7** | Ac-KYIGSRY | PAAA | Laminin β1 chain | 76 kDa Laminin binding protein (Glycosaminoglycan) α3β1, α4β1, α6β1 |
| **8** | Ac-KGACRRETAWACGA | PAAA | Phage display library | Integrin α_5_β_1_ |
| **9** | Ac-KGCWLDVCGY | PAAA | Fibronectin CS-1 domain | Integrin α_4_β_1_ |
| **10** | Ac-KSVVYGLR | PAAA | Osteopontin | Integrin α4β1, α4β7, α9β1, αvβ3 |
| **11** | Ac-KVDTYDGRGDSVVYGLR | PAAA | Osteopontin | Integrin α4β1, α4β7, α9β1, αvβ3 |
| **12** | Ac-KYGYYGDALR | PAAA | Laminin-1 | Integrin α2β1 |
| **13** | Ac-KFYFDLR | PAAA | Collagen IV | Integrin α2β1 |
| **14** | K(N3)-IKVAV | PAPA | Laminin α-1 chain | Integrin α3β1, α6β1, α4β1 |
| **15** | Ac-KDGEA | PAAA | Collagen I | Integrin α2β1 |
| **16** | K(N3)GDITYVRLKF | PAPA | Laminin γ1-chain | Integrin αvβ3 |
| **17** | Ac-KGDIRVTLNRL | PAAA | Laminin γ1-chain | Unknown/not integrin |
| **18** | K(N3)GFQVAYIIIKA | PAPA | Laminin α1 chain | Heparan sulphate proteoglycan/β1 Integrins |
| **19** | Ac-KGSIYITRF | PAAA | Laminin α1 chain | Heparan sulphate proteoglycan |
| **20** | K(N3)GLSIELVRGRVKV | PAPA | Laminin α1 chain G | Heparan sulphate proteoglycan |
| **21** | Ac-KGLQVQLSIR | PAAA | Laminin α1 chain | Syndecans |
| **22** | Ac-KGHQNQMDYATLQLQ | PAAA | Laminin α1 chain | Integrin α2β1 |
| **23** | K(N3)GSYNGIIFFLK | PAPA | Laminin α5 chain G | Heparan sulphate proteoglycan |
| **24** | Ac-KGHQMNGSVNVSVG | PAAA | Laminin α5 chain G | Heparan sulphate proteoglycan |
| **25** | Ac-KGSYLQFVGI | PAAA | Laminin α5 chain G | Heparan sulphate proteoglycan |
| **26** | Ac-KGAPVNVTASVQIQ | PAAA | Laminin α5 chain G | Heparan sulphate proteoglycan |
| **27** | Ac-KGAFGVLALWGTR | PAAA | Laminin β1 chain | Heparan sulphate proteoglycan/Integrins |
| **28** | K(N3)GDSITKYFQMSL | PAPA | Laminin β1 chain | Heparan sulphate proteoglycan |
| **29** | Ac-KGILQQSAADIAR | PAAA | Laminin β1 chain | Heparan sulphate proteoglycan/Integrins |
| **30** | K(N3)GTSIKIRGTYS | PAPA | Laminin γ1 chain | Integrin α2β1 |
| **31** | K(N3)GGKKQRFRHRNRKG | PAPA | Vitronectin | Heparin binding site |
| **32** | K(N3)GFHRRIKA | PAPA | Bone Sialoprotein | Heparin binding site |
| **33** | Ac-KGGWQPPRARI | PAAA | Fibronectin HBS | Heparan sulphate proteoglycan |
| **34** | Ac-KGGNGEPRGDTYRAY | PAAA | Bone Sialoprotein | Integrins |
| **35** | Ac-KGGPQVTRGDVFTM-OH | PAAA | Vitronectin | Integrins |
| **36** | cRGDfK | PAAA/ PAPA | Synthetic | Integrins |

**Supporting Information Table S2: This table describes the chemical properties that are related to each solubility of each peptide listed in Supporting Information Table S1. The number of residues, isoelectric point, charge, and hydrophobicity is included for each peptide. Isoelectric point, charge and hydrophobicity were calculated using the tool at** [**http://www.lifetein.com/peptide-analysis-tool.html**](http://www.lifetein.com/peptide-analysis-tool.html)**. This tool was unable to account for the presence of azide groups or peptide cyclization so these factors were not included in this analysis. The peptides highlighted in bold dissolved incompletely.**

| Peptide # | # of residues | Isoelectric point | Charge | Hydrophobicity at pH 2.0 | Hydrophobicity at pH 6.8 |
| --- | --- | --- | --- | --- | --- |
| 1 | 10 | 10.72 | +3 Basic | 24.6 | 30.1 |
| 2 | 12 | 10.02 | +1 Basic | 24.67 | 20.83 |
| 3 | 9 | 11.4 | +3 Basic | 19.11 | 24.22 |
| 4 | 13 | 11.86 | +4 Basic | 10.46 | 13.92 |
| 5 | 13 | 10.7 | +4 Basic | 1.08 | 8.92 |
| 6 | 6 | 11.51 | +3 Basic | -33.17 | -20.17 |
| 7 | 7 | 9.85 | +2 Basic | 18.29 | 26.14 |
| 8 | 14 | 8 | +2 Basic | 22 | 20.71 |
| 9 | 10 | 3.1 | Neutral | 36.1 | 35.6 |
| 10 | 8 | 10.32 | +2 Basic | 39.62 | 34.12 |
| 11 | 17 | 4.27 | Neutral | 17.53 | 14.47 |
| 12 | 10 | 6.78 | +1 Basic | 21.3 | 23.8 |
| 13 | 7 | 6.95 | +1 Basic | 36 | 37.86 |
| 14 | 7 | 10.72 | +2 Basic | 32 | 34.43 |
| 15 | 5 | 2.98 | -1 Acidic | 0 | -13.6 |
| **16** | **11** | **10.3** | **+2 Basic** | **28.64** | **30.27** |
| 17 | 11 | 11.29 | +2 Basic | 22.18 | 21.91 |
| **18** | **12** | **10.31** | **+2 Basic** | **43.5** | **46.58** |
| 19 | 9 | 10.32 | +2 Basic | 31.56 | 36.56 |
| 20 | 14 | 11.56 | +3 Basic | 29.43 | 29.79 |
| 21 | 10 | 11.51 | +2 Basic | 27.3 | 31.3 |
| 22 | 15 | 5 | +1 Basic | 11.53 | 16 |
| **23** | **12** | **10.31** | **+2 Basic** | **34.25** | **38.58** |
| **24** | **14** | **10.28** | **+2 Basic** | **8.43** | **13.21** |
| 25 | 10 | 6.09 | +1 Basic | 35.8 | 39.7 |
| **26** | **14** | **6.41** | **+1 Basic** | **19.79** | **20.5** |
| 27 | 13 | 11.51 | +2 Basic | 38.38 | 40.62 |
| 28 | 13 | 9.64 | +1 Basic | 23.28 | 25 |
| 29 | 13 | 7.13 | +1 Basic | 24.38 | 23.38 |
| **30** | **12** | **10.83** | **+3 Basic** | **13.42** | **18.08** |
| 31 | 15 | 12.98 | +9 Basic | -17.4 | -6.27 |
| 32 | 9 | 12.53 | +5 Basic | 7.89 | 19 |
| 33 | 11 | 12.51 | +3 Basic | 2.91 | 7.64 |
| 34 | 15 | 7 | +1 Basic | -1.87 | -2.93 |
| 35 | 14 | 7.13 | +1 Basic | 14.64 | 14.36 |
| 36/RGDfK | 5 | 10.27 | +1 Basic | 2.2 | 1 |
| RADfK | 5 | 10.27 | +1 Basic | 11.6 | 9.2 |

**
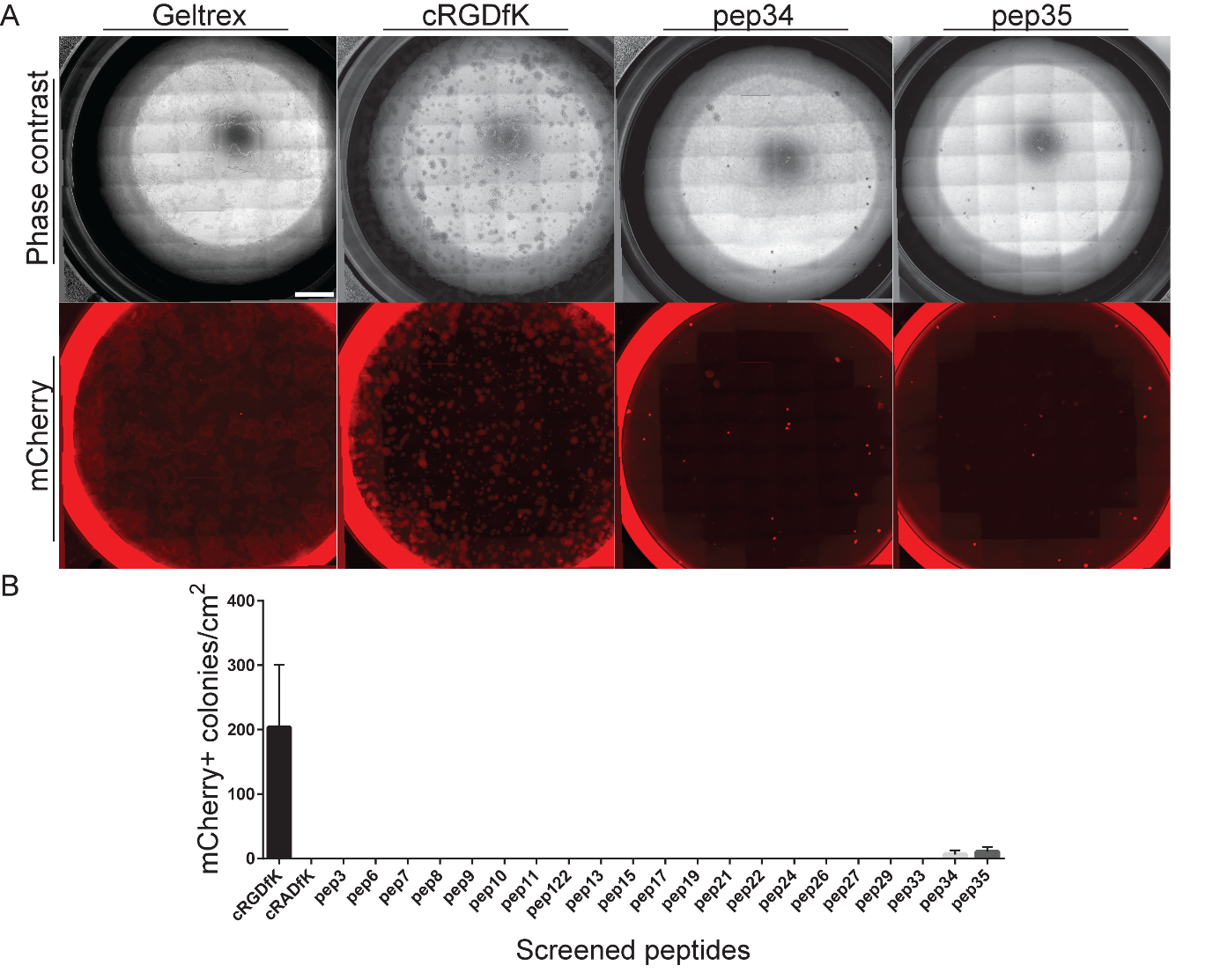
**

**Supporting Information Figure S1: Adhesion of H9-OCT4*^2AChryIM/w^* cells to peptide-modified PAAA-40UV coatings.** H9-*OCT4^2AChryIM/w^* cells were seeded at a density of 15 000 cells/cm^2^ in E8 medium in wells that had been coated with Geltrex™ or PAAA coatings modified with solutions containing 200 μM of peptide cRGDfK or test peptides. Wells were scanned 48 hours after seeding and colonies were counted. **(A)** Representative scans of whole wells coated with Geltrex, or PAAA coatings modified with hit peptides. Scale bar represents 2 mm in all images. **(B)** Colony counts from the H9-*OCT4^2AChryIM/w^* adhesion assay. The mean number of colonies/cm^2^ are presented from three independent experiments. Error bars represent standard deviations from the mean.

**
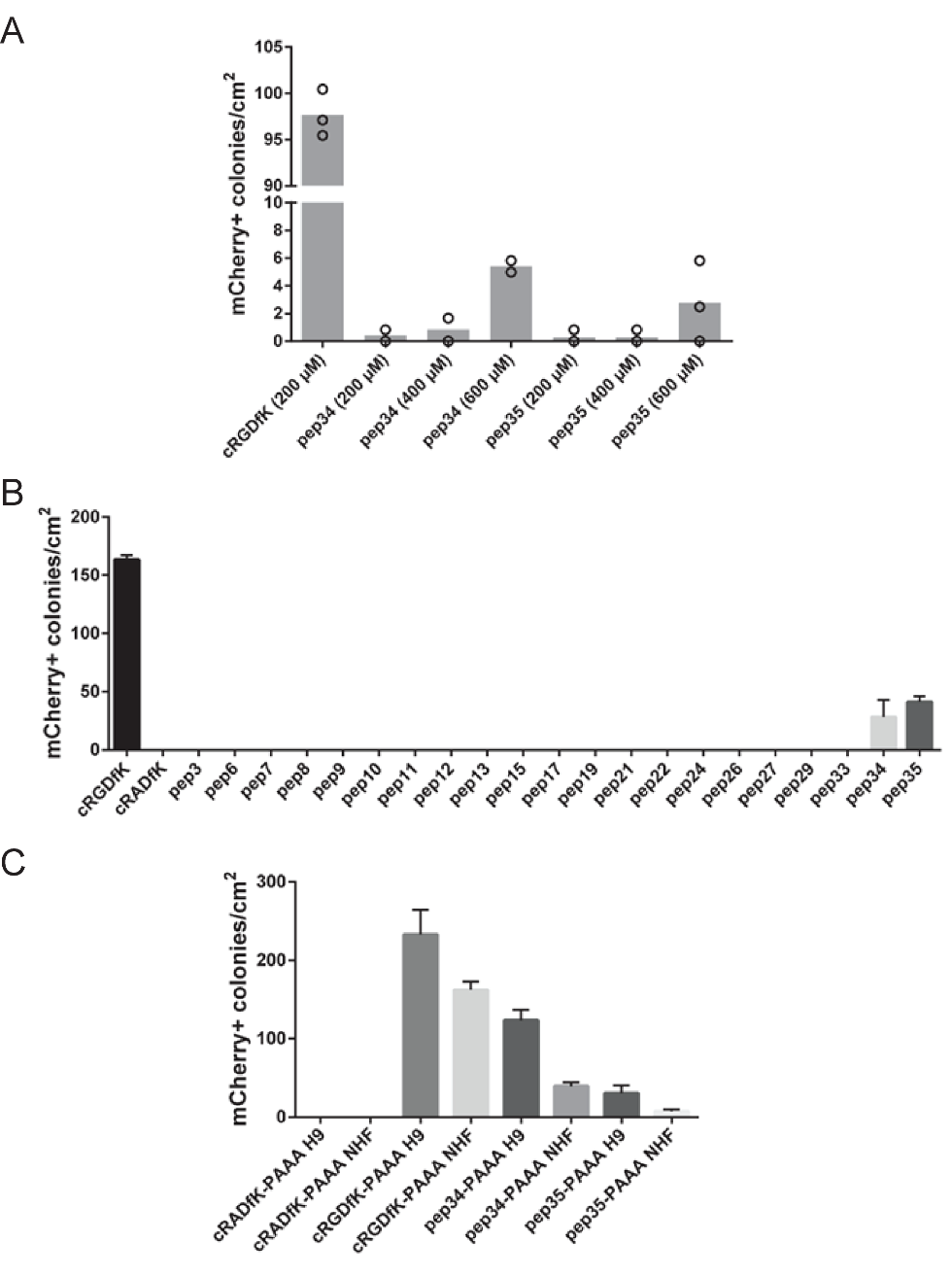
**

**Supporting Information Figure S2: Optimisation of cRGDfK-PAAA coatings for hPSC adhesion. (A)** A H9-*OCT4^2AChryIM/w^* adhesion assay was performed on PAAA surfaces modified with high concentrations of the candidate peptides, with potential for maintaining hPSC cultures, 34 (pep34) and 35 (pep35). PAAA-coated wells were modified with solutions containing 200 μM, 400 μM or 600 μM of pep34 or pep35 and were seeded with 15 000 H9-*OCT4^2AChryIM/w^* cells/cm^2^ in E8 medium. Control wells were modified with solutions containing 200 μM of cRGDfK peptide. Scanned images were captured of all wells 48 hours after seeding and mCherry^pos^ colonies were counted. Means of colony counts are presented from two or three technical replicates and individual data points are marked with hollow circles. **(B)** An H9-*OCT4^2AChryIM/w^* adhesion assay in a single experiment that re-screened peptide-modified PAAA coatings using the thinner PAPA-25UV surfaces. PAAA-25UV-coated wells were modified with solutions containing 200 μM of each peptide and were seeded with 15 000 H9-*OCT4^2AChryIM/w^* cells/cm^2^ in E8 medium. Negative control wells were modified with solutions containing 200 μM of the non-binding cRADfK peptide. Scanned images were captured of all wells 48 hours after seeding and mCherry^pos^ colonies were counted. Means of three technical replicates are presented. Error bars represent standard deviations from the mean. **(C)** HPSC adhesion to PAAA coatings modified with cRGDfK, peptide 34 (pep34) and peptide 35 (pep35) was validated using H9 and NHF-1-3 (NHF) hPSCs. PAAA-40UV coated surfaces were modified with solutions containing 200 μM of cRGDfK, pep34 or pep35. Wells were seeded with H9 or NHF-1-3 (NHF) hPSCs at a density of 15 000 cells/cm^2^. Colony counts from phase contrast scans of these wells compared to non-binding cRADfK-modified control wells. Means of three technical replicates are presented. Error bars represent standard deviation.

**
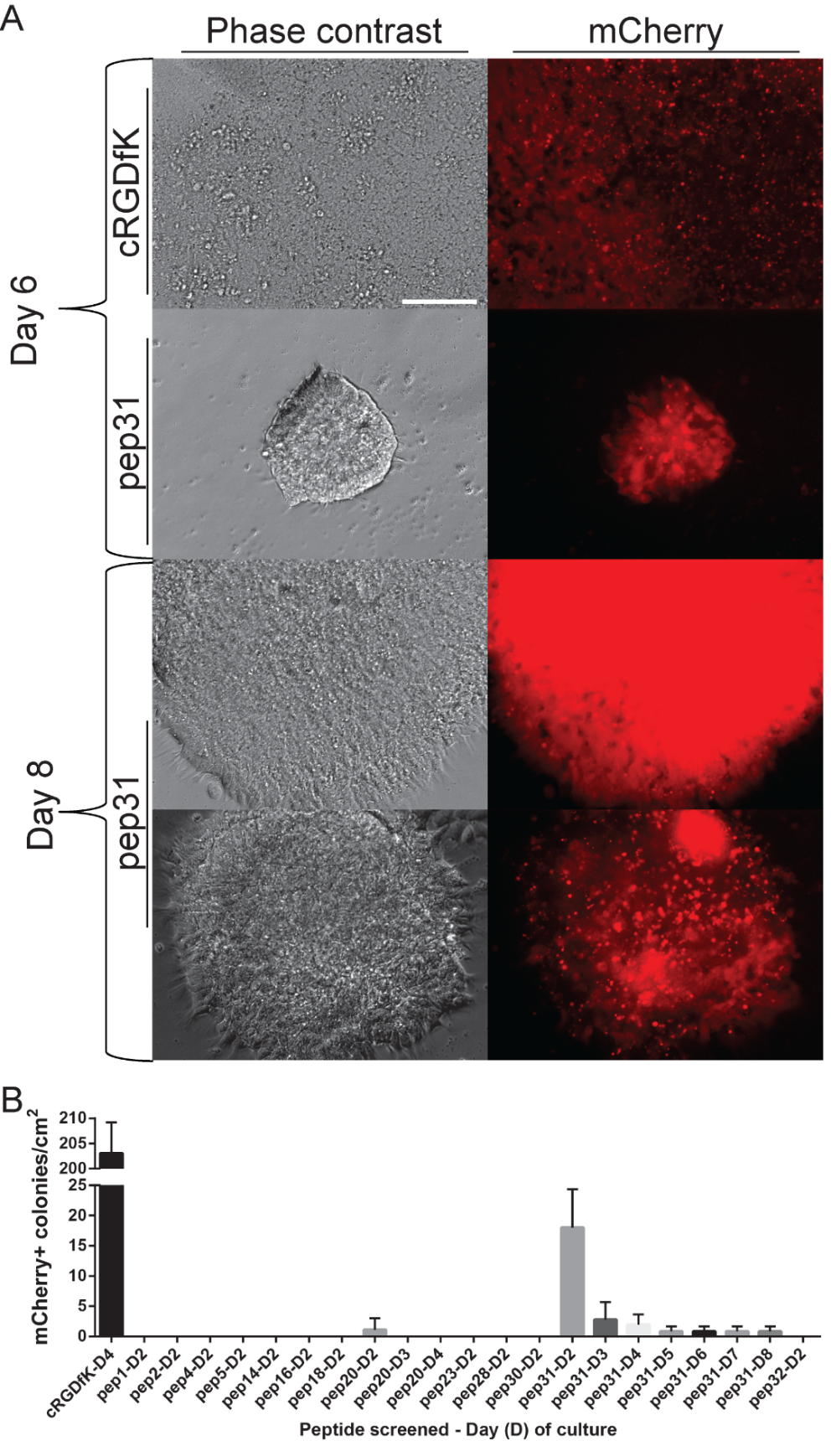
**

**Supporting Information Figure S3: Screening of hPSC adhesion to peptide-modified PAPA-coated surfaces.** H9-*OCT4^2AChryIM/w^* cell adhesion to peptide-modified PAPA-30UV-coatings. Quantification of adherent colonies was performed from day 2 (D2) out to day 8 (D8) in some cases. **(A)** H9-*OCT4^2AChryIM/w^* mCherry cells attached to cRGDfK-PAPA formed large and mostly mCherry^pos^ colonies. At the same time point the colonies on pep31-PAAA were considerably smaller and more rounded than those on cRGDfK-PAPA. By the eighth day of culture H9-*OCT4^2AChryIM/w^* colonies on pep31-PAPA colonies had grown considerably and were observed to contain fine neural-like projections while remaining mostly mCherry-positive or to be mostly mCherry-negative and show more distinct morphological differentiation. Scale bar represents 100 μm on all images. **(B)** Colony counting results indicate that the number of hPSC colonies on pep20-PAPA and pep31-PAPA decreased after day 2, while high numbers of mCherry^pos^ colonies (>200 colonies/cm^2^) remained adherent to PAPA-cRGDfK out to day 4. Means of three technical replicates are presented. Error bars represent standard deviations from the mean.


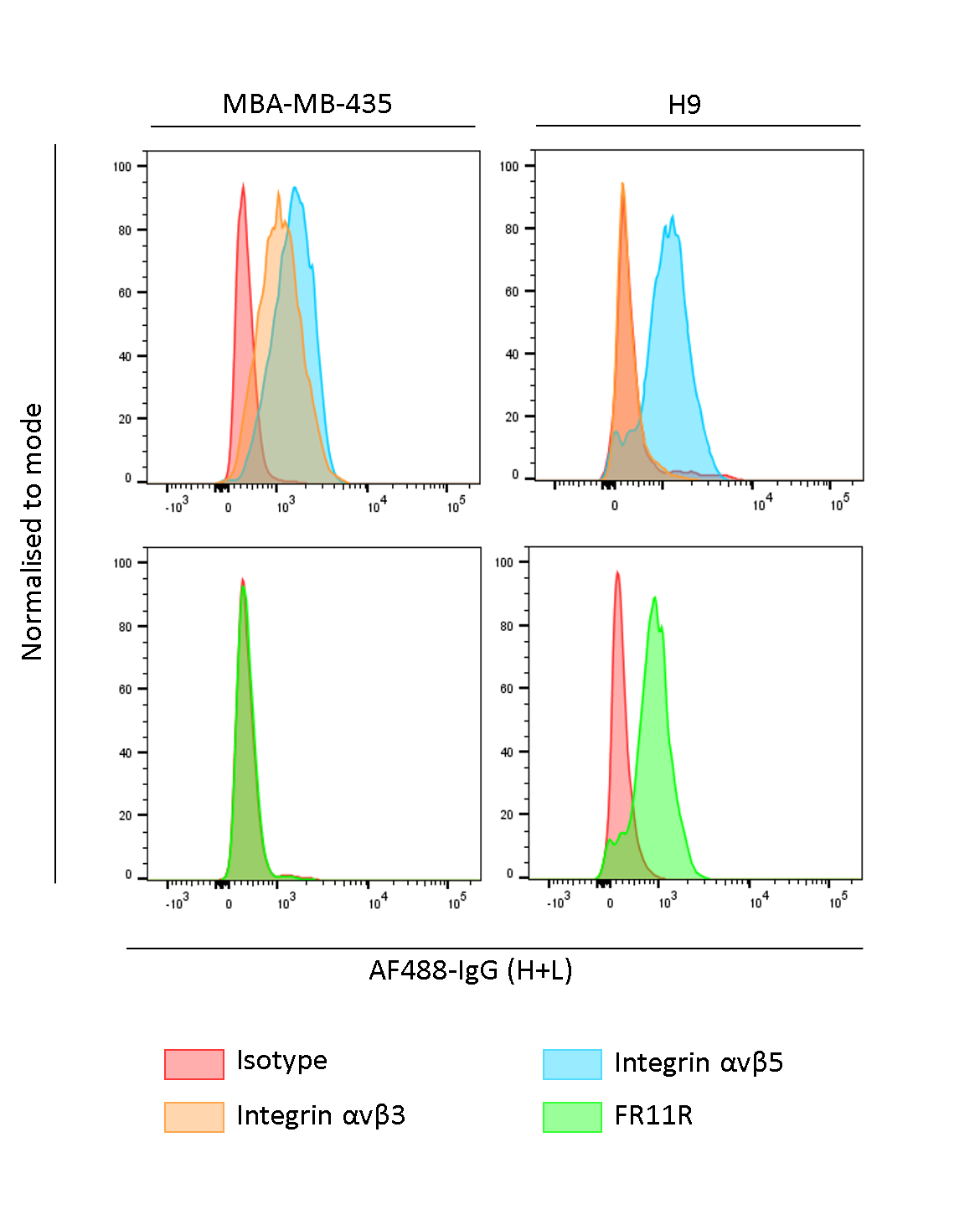


F11R

**Supporting Information Figure S4: Flow cytometric staining of H9 hPSCs and MDA-MB-435 cells for integrin αvβ3 and αvβ5.** MDA-MB-435 cells (left), but not H9 hPSCs (right) stained positive for αvβ3 integrin (orange) while both cell lines were positive for αvβ5 integrin (blue) compared to isotype controls (red). Cells were also stained with the pluripotency marker F11R (green), where H9 cells exhibited a similar staining pattern to αvβ5.

**
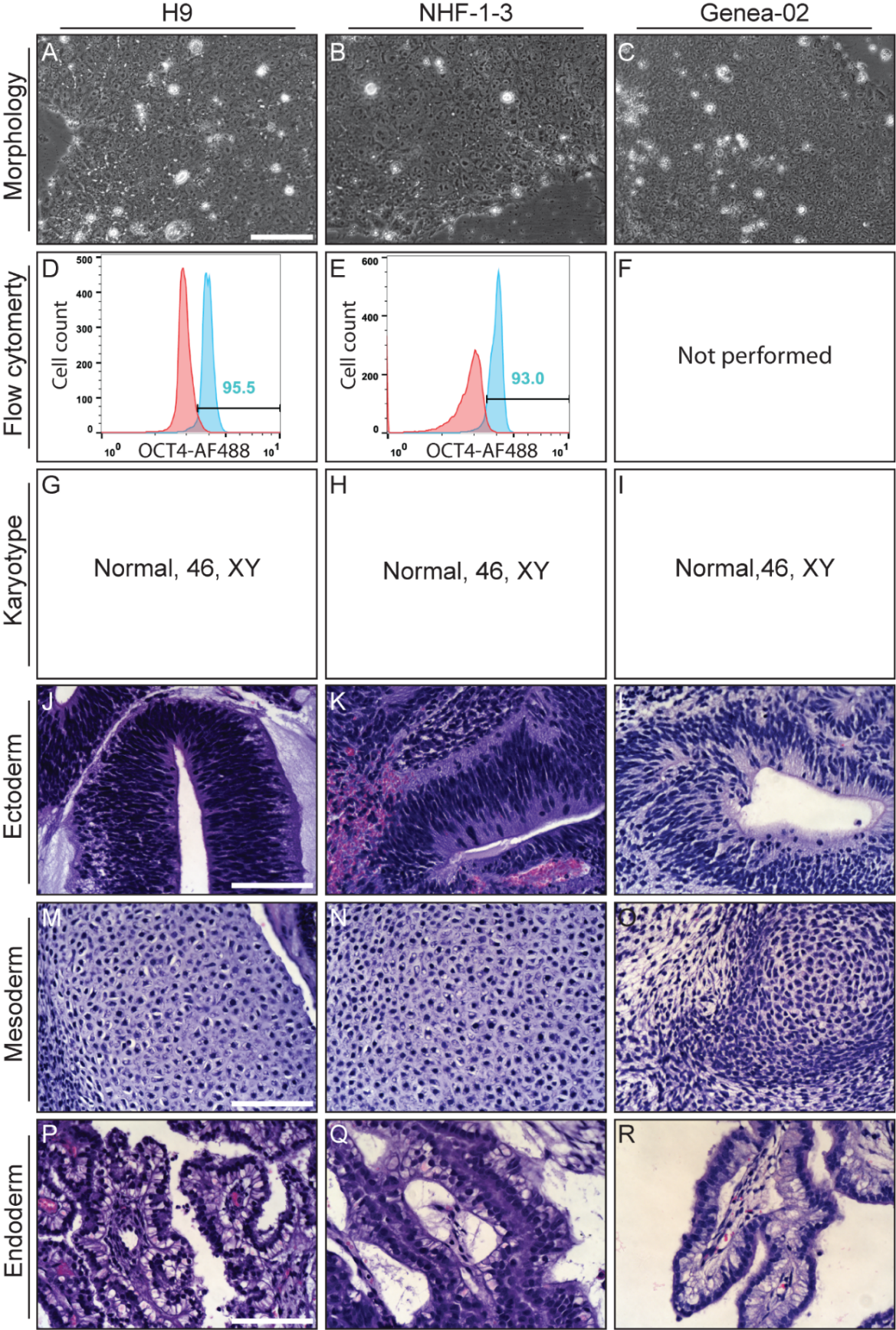
**

**Supporting Information Figure S5: Characterisation of starter hPSC cultures adapted to E8/Geltrex™ culture conditions.** (A-C) Phase contrast images were captured at day 3 post-seeding of cultures of H9, NHF-1-3 and Genea-02 cultures adapted to culture in E8 medium on Geltrex™-coated TCPS. (D-F) Representative histogram plots show flow cytometric detection of OCT4 (AF488) in H9 and NHF-1-3 cells compared to cells stained with an isotype control antibody. The percentage of cells in which OCT4 was detected are included in each plot. (G-I) G-banding karyotype results of H9, NHF-1-3 and Genea-02 hPSC cultures maintained in E8 medium on Geltrex™-coated surfaces. For chromosome spreads please see Supplementary Figure 11 (J-R) Teratoma formation assay results are also presented for the three adapted hPSC lines, showing formation of (J-L) neural rosettes (ectoderm), (M-O) cartilage (mesoderm) and (P-R) secretory epithelial cells (endoderm). Scale bars, 100 μm.

**
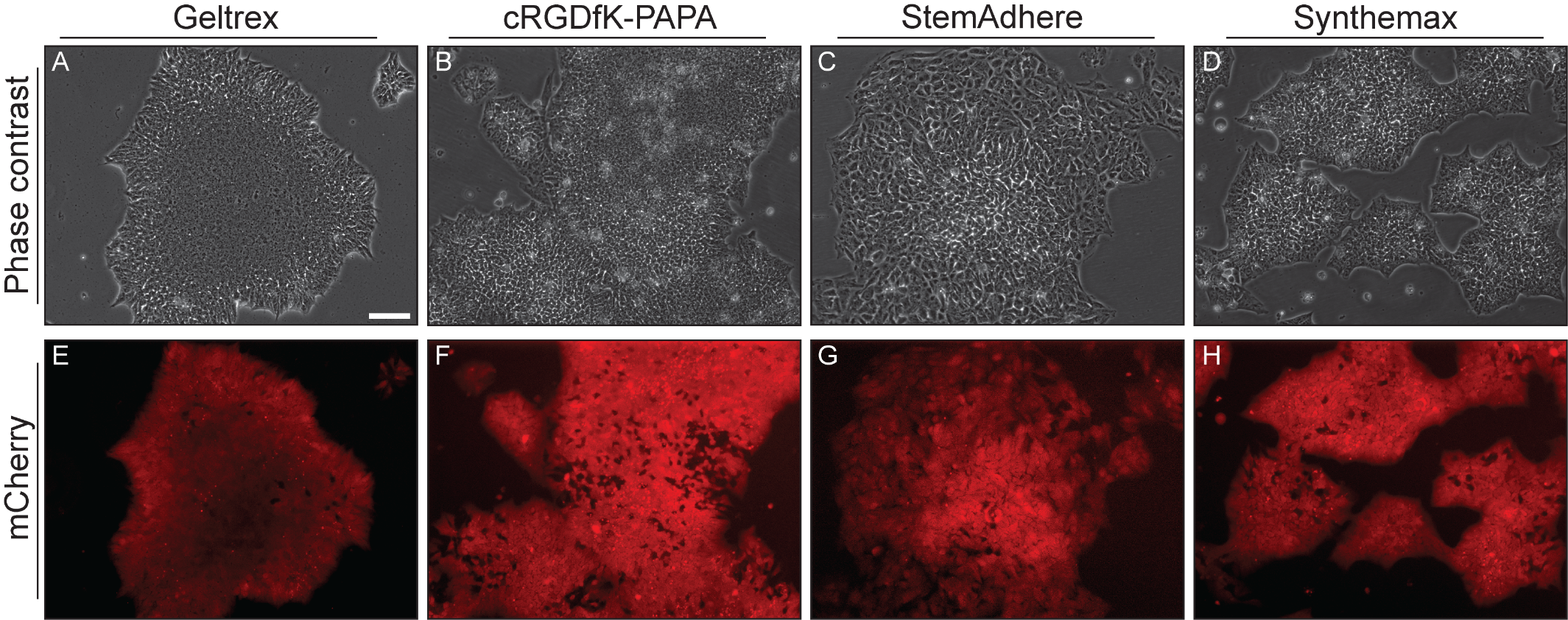
**

**Supporting Information Figure S6: Morphological and fluorescent assessment of H9-OCT4*^2AChryIM/w^* cultures maintained for 3 days in E8 medium on test surfaces.** (A-D) Phase contrast and (E-L) mCherry fluorescence photomicrographs are presented of H9-*OCT4^2AChryIM/w^* cells three days post-seeding, following two serial passages in flasks coated with (A,E) Geltrex™, (B,F) cRGDfK-PAPA, (C,G) StemAdhere™ and (D,H) Synthemax™. All fluorescence images were acquired with 600 ms exposure times. The mCherry signal in image G was less bright than images E, F and H and was not visible when all images were treated identically. Image G was therefore scaled to a grey level minimum of 370 and maximum of 580 instead of the same grey level range (1000 to 2000) as E, F and H. Scale bar represents 100 μm on all images.

**
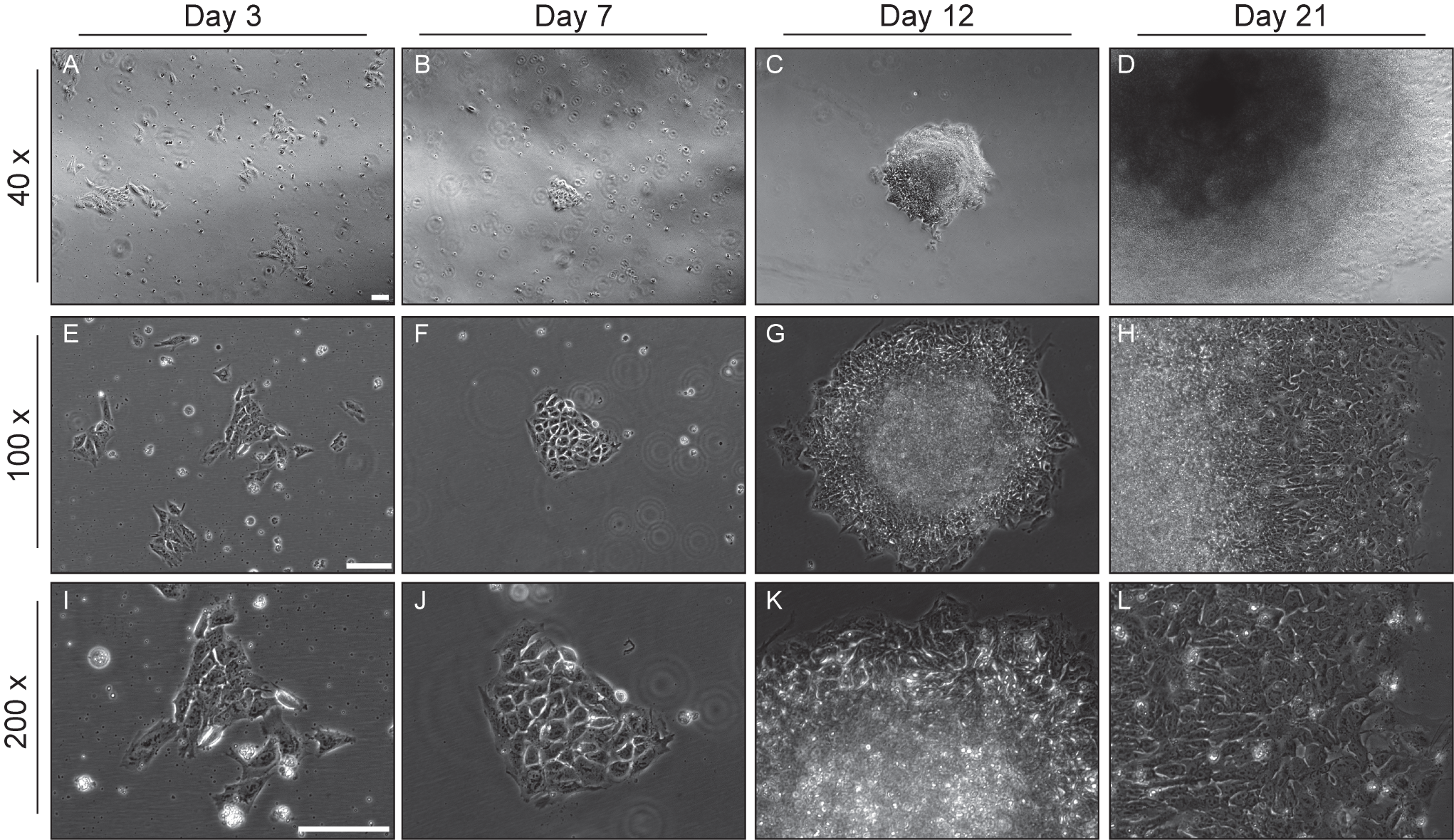
**

**Supporting Information Figure S7: The gradual recovery of Genea-02 cells from the first passage on StemAdhere™-coated flasks**. Representative images are presented from days 3, 7, 12 and 21 of culture, captured at 40x, 100x and 200x magnification. Scale bars represent 100 μm and apply to every image in each row.

**
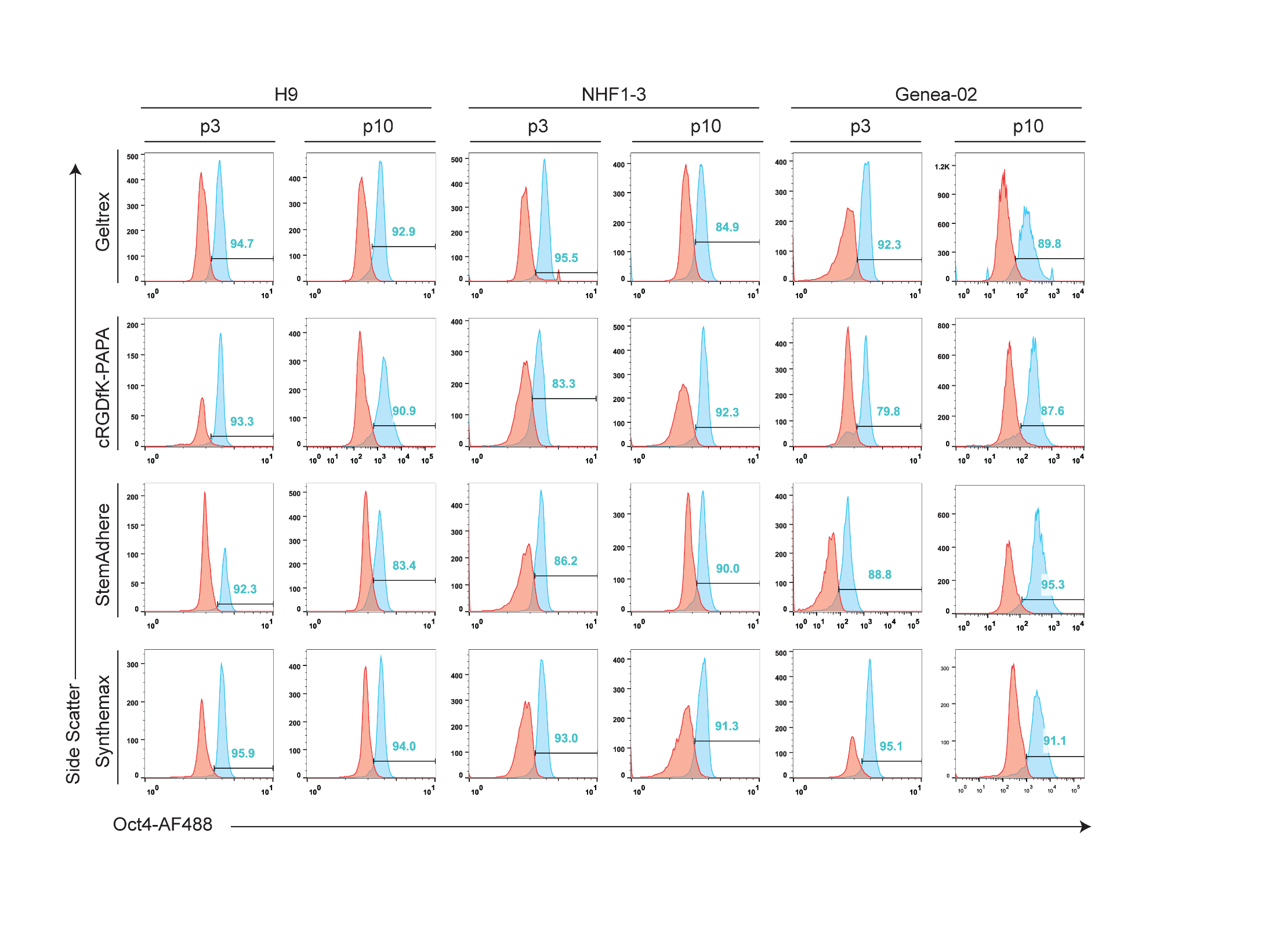
**

**Supporting Information Figure S8: Flow cytometric assessment of OCT4 protein detected in hPSCs maintained on test culture surfaces.** After cultures of H9, NHF-1-3 and Genea-02 hPSCs had been maintained for 3 and 10 passages in E8 medium in flasks coated with Geltrex™, cRGDfK-PAPA, StemAdhere™ or Synthemax™, the cells were immunostained for OCT4 and assessed by flow cytometry. Gates for AF488-OCT4 were set against cells stained with the IgG1 isotype control antibody (red) and applied to samples that had been immunostained for OCT4 (blue). The percentage of OCT4-AF488-stained cells are displayed on each plot. The different axis scales are an artefact of samples being analysed on different flow cytometers.

**
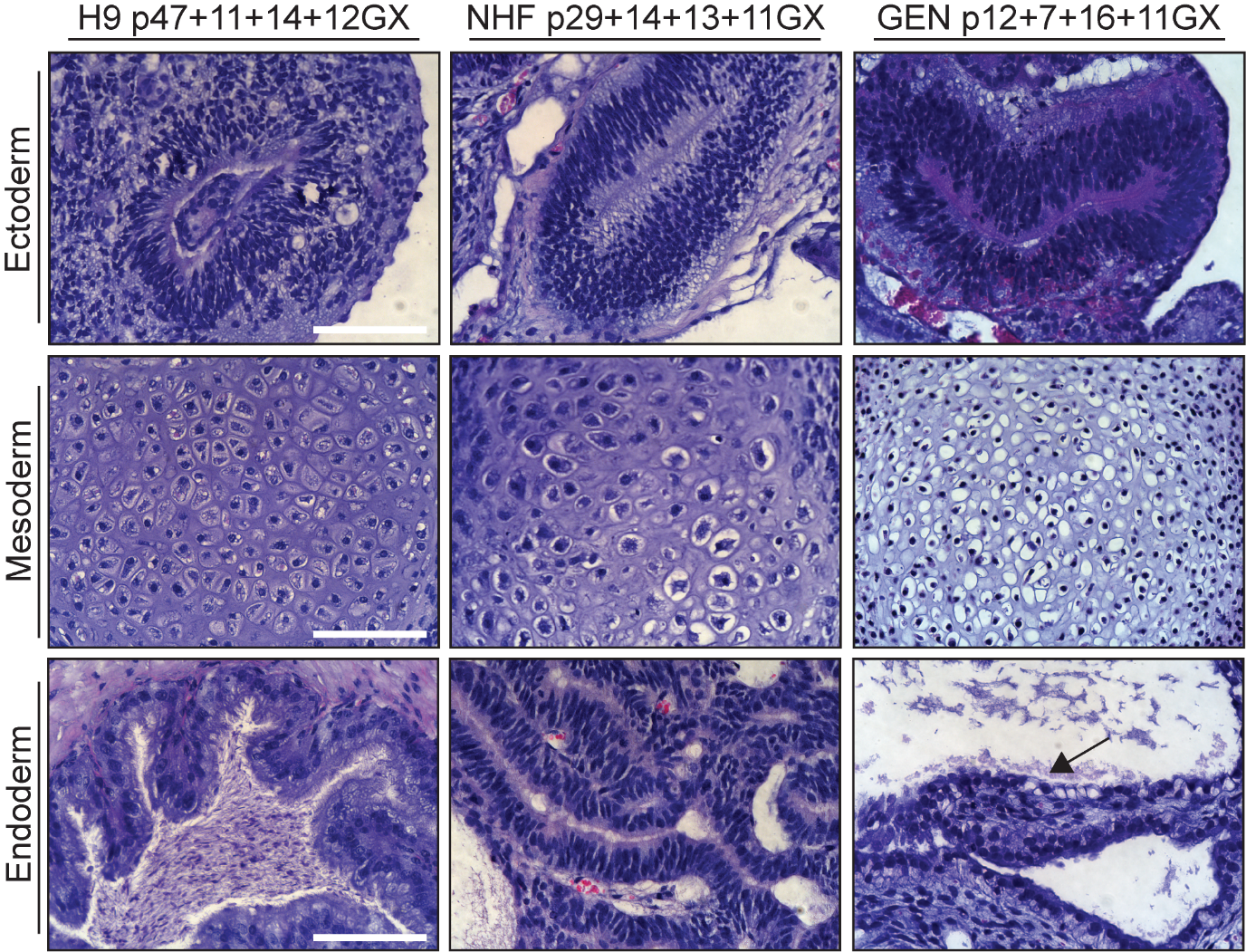
**

**
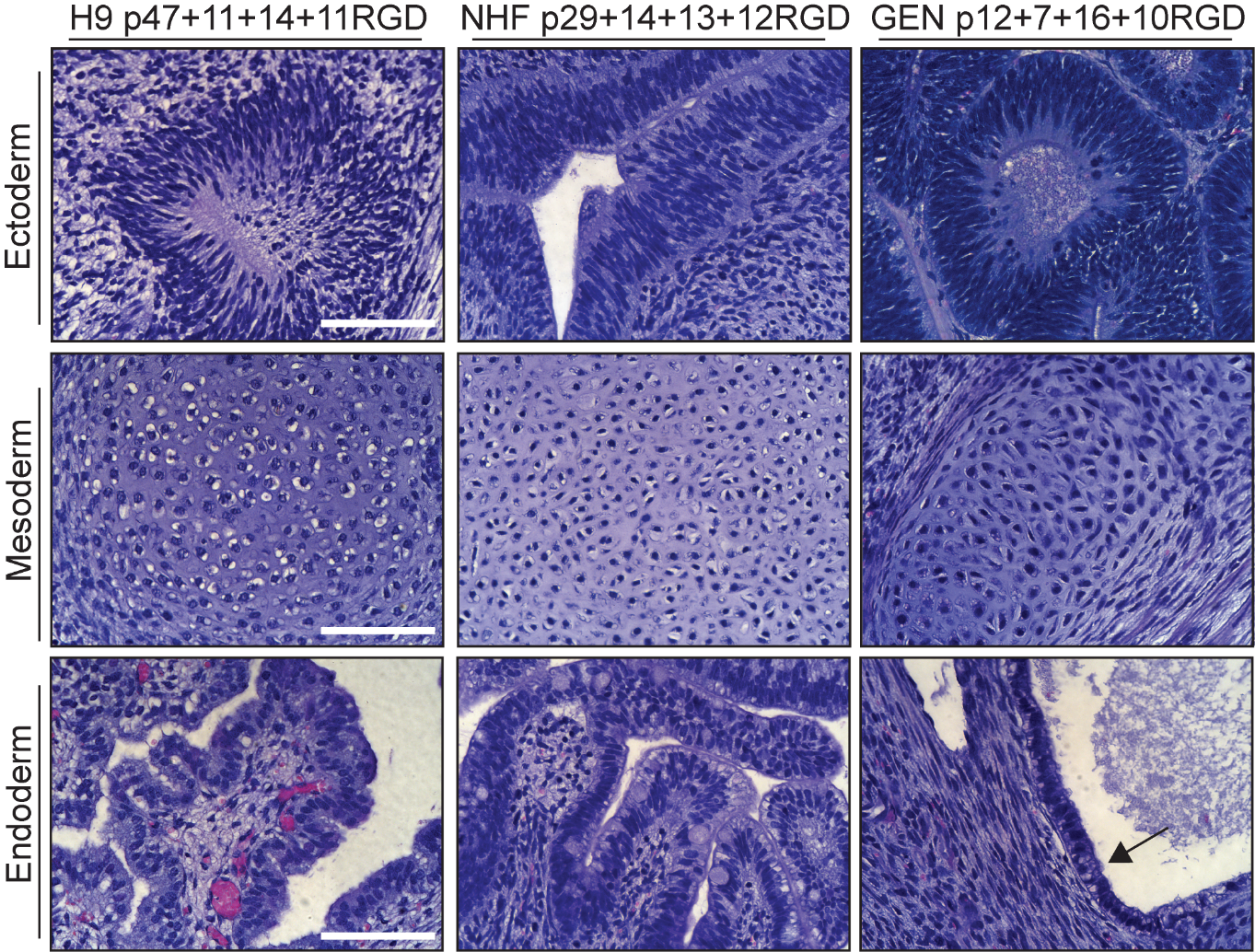
**

**Supporting Information Figure S9** Caption is on the following page.

**
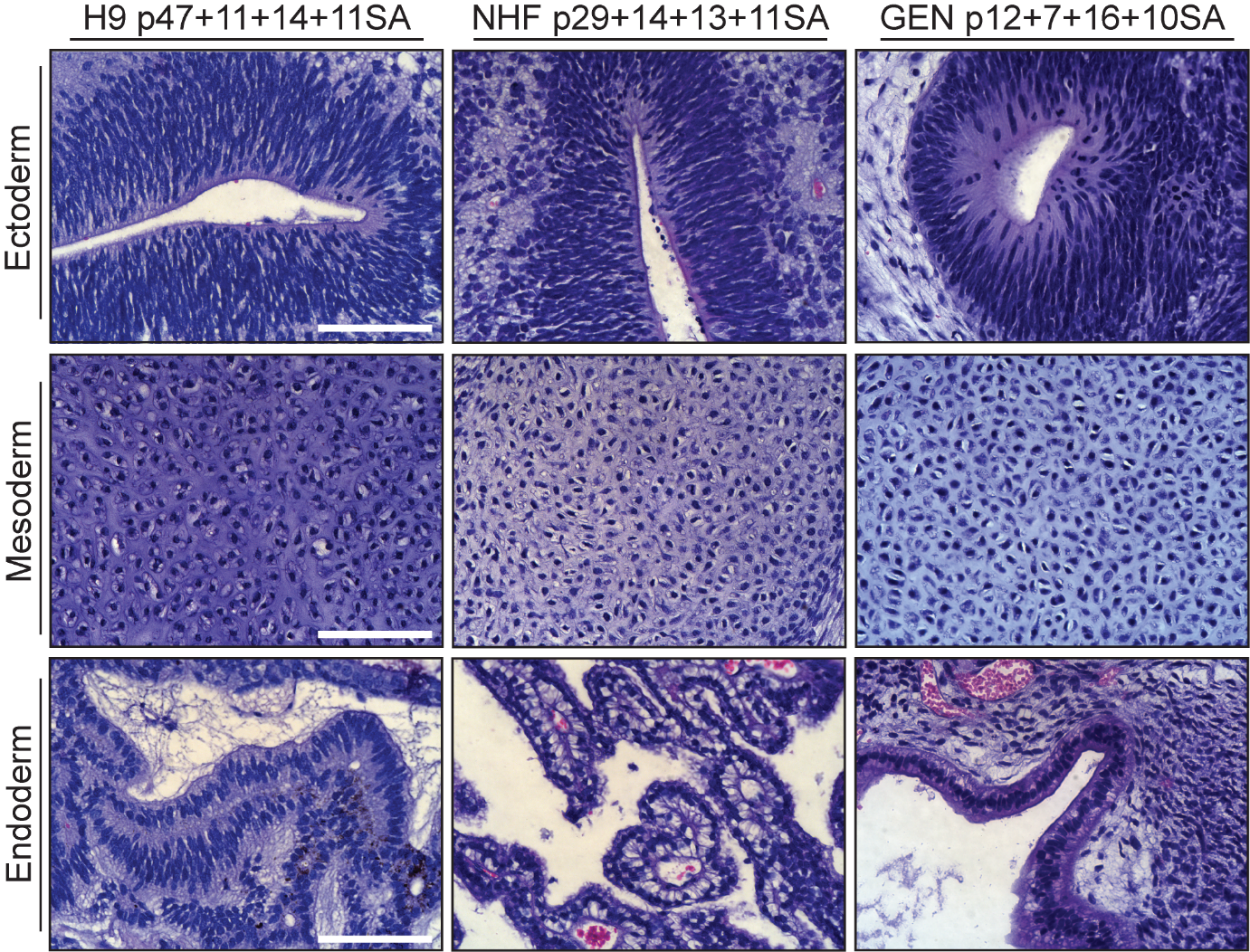
**

**
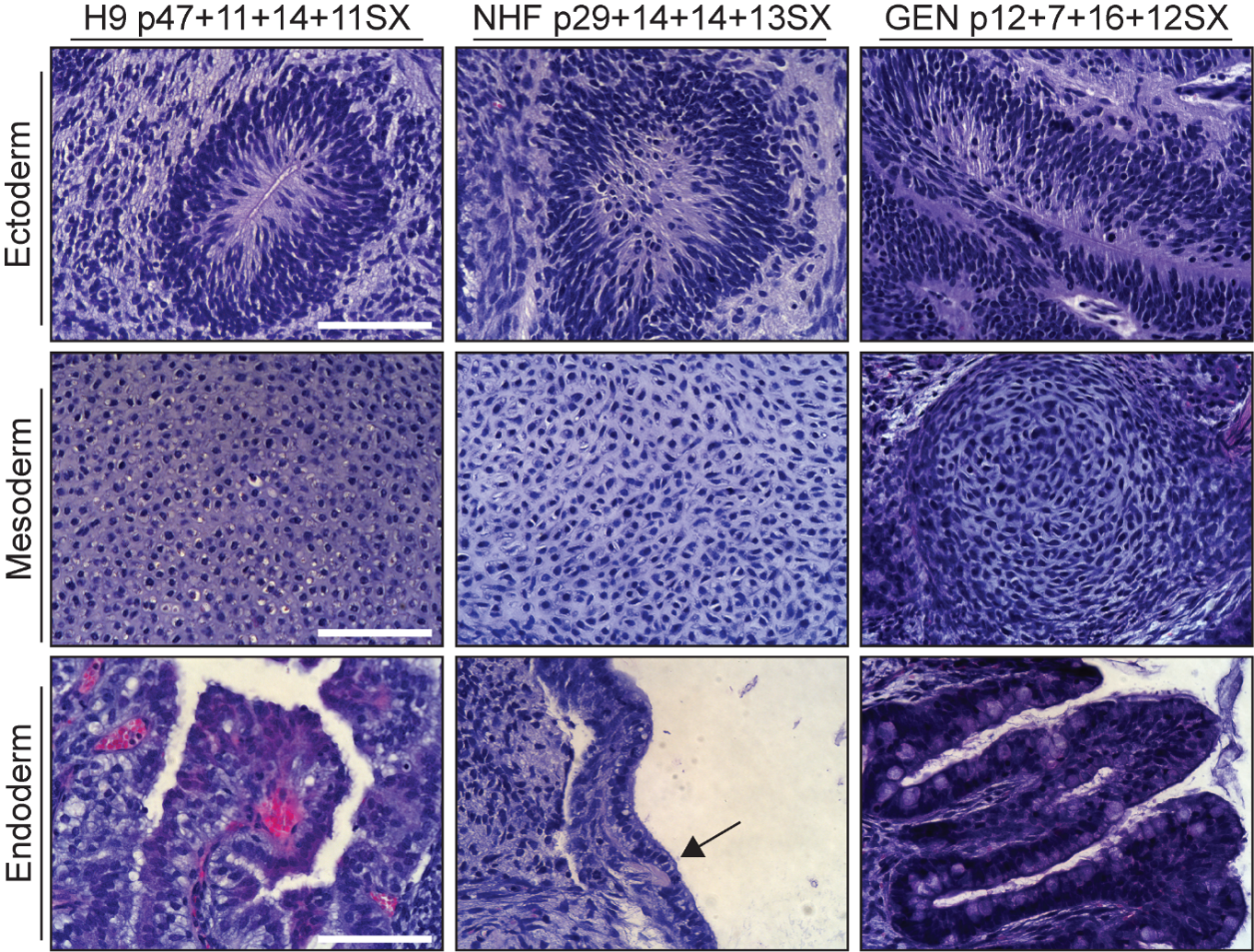
**

**Supporting Information Figure S9: The results of teratoma formation assays for hPSCs following long-term maintenance on test culture surfaces.** H9, NHF-1-3 (NHF) and Genea-02 (GEN) cultures that had been maintained for at least 10 passages in flasks coated with Geltrex™ (GX), cRGDfK-PAPA (RGD), StemAdhere™ (SA) or Synthemax™ (SX) were injected into the testis capsules of immunocompromised mice and formed tumours. Representative images are shown for sections of those tumours which had been stained with haematoxylin and eosin, and scored for the presence of tissue types representative of the three germ layers including neural rosettes (ectoderm), cartilage (mesoderm) and secretory epithelial cells (endoderm, indicated by arrows for GEN GX, GEN RGD and NHF SX). Scale bars represent 100 μm in all images.

**
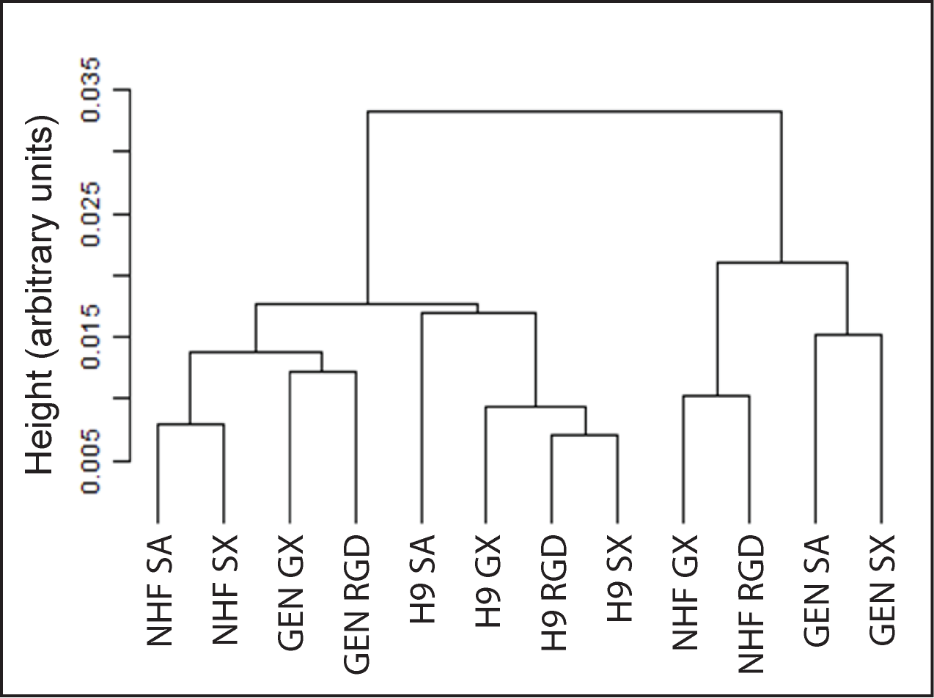
**

**Supporting Information Figure S10: PluriTest™ phylogenetic tree.** The PluriTest™ assay was performed on H9, NHF-1-3 (NHF) and Genea-02 (GEN) cultures that had been maintained for 10 passages in flasks coated with Geltrex (GX), cRGDfK-PAPA (RGD), StemAdhere™ (SA) or Synthemax™ (SX). After the samples were transformed with a variance stabilising transformation a phylogenetic tree was generated representing the relationships between data sets.


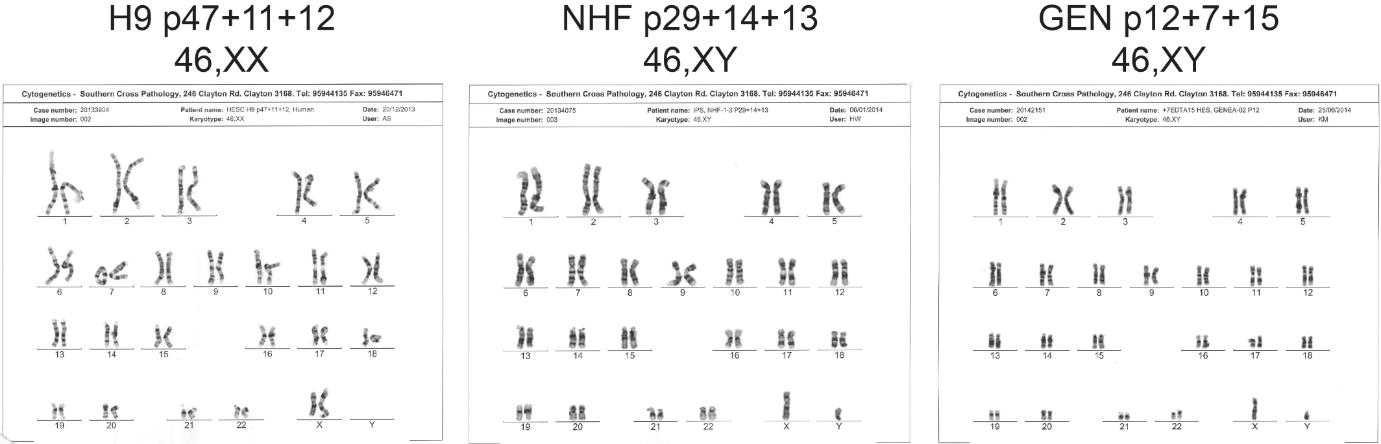


**Supporting Information Figure S11:** Karyograms are presented from G-banding karyotype analyses of H9, NHF-1-3 (NHF) and Genea-02 (GEN) hPSCs that had been adapted to culture on Geltrex™-coated surfaces in E8 medium for 12, 13 and 15 passages, respectively.


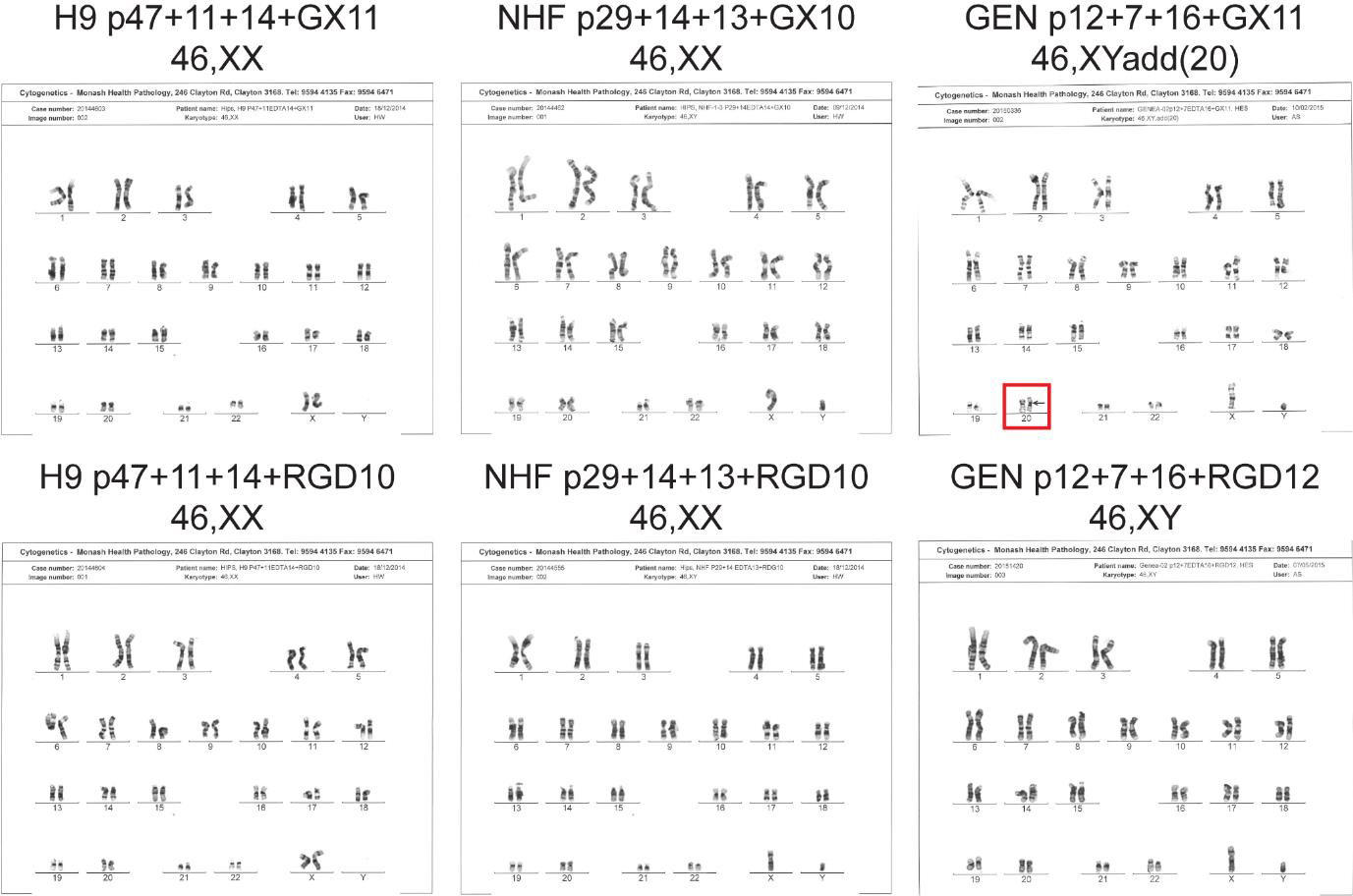


**Supporting Information Figure S12**: G-banding karyotyping assessment of hPSC cultures maintained on defined culture surfaces. Karyograms are presented from cultures of H9 (left column), NHF-1-3 (NHF, central column) and Genea-02 (GEN, right column) hPSCs that had been maintained for at least 10 passages in flasks coated with Geltrex™ (GX), cRGDfK-PAPA (RGD), StemAdhere™ (SA) or Synthemax™ (SX). Red boxes highlight karyotypic abnormalities.


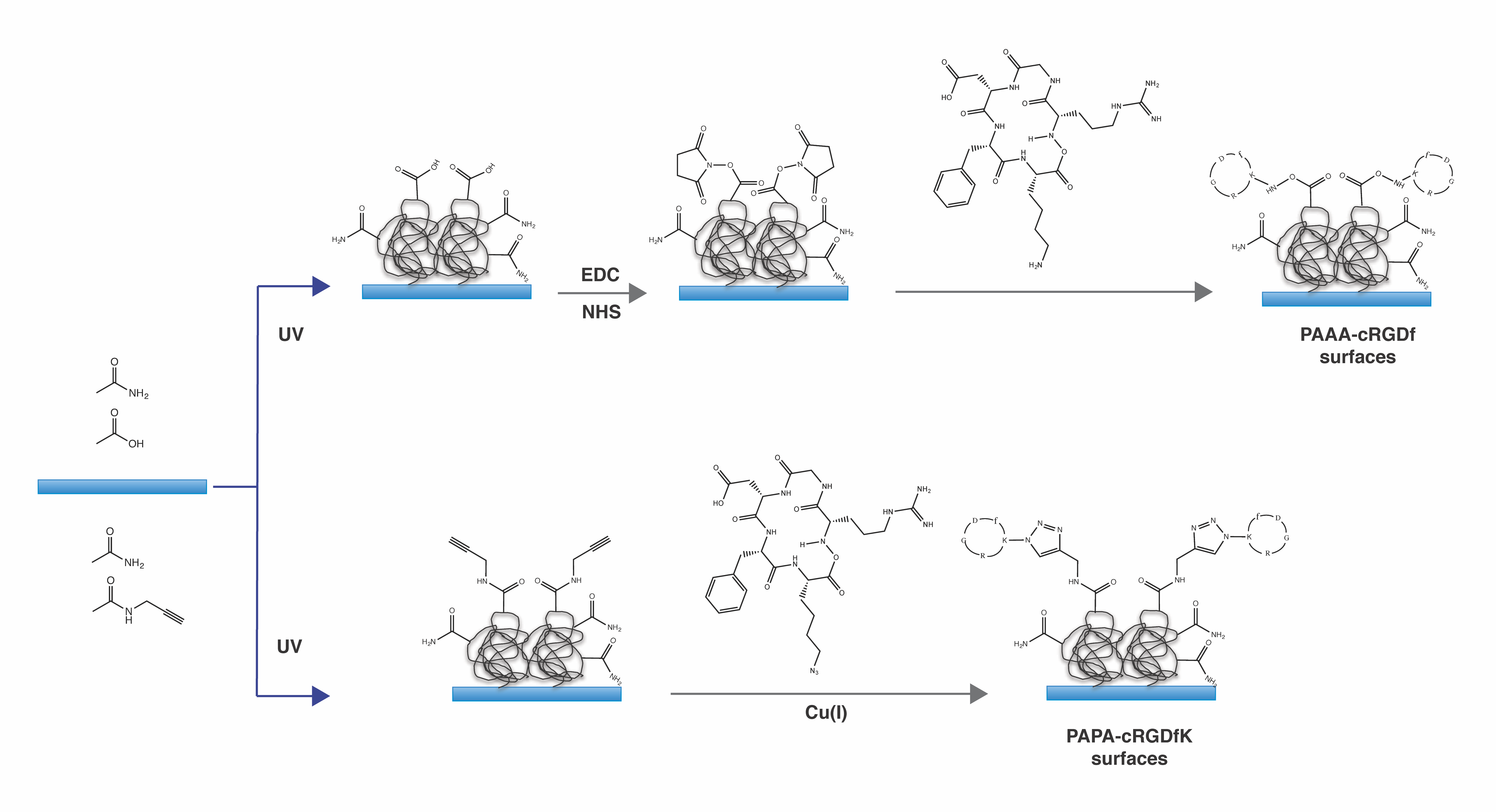
**Supporting Information Figure S13**: Schematic showing preparation of PAAA (upper) and PAPA surfaces (lower). For the preparation of PAAA surfaces (upper), copolymer coatings formed from acrylamide and acrylic acid monomers containing carboxylic acids are activated using EDC and NHS, to form active NHS esters, which then react with the amine side chain of the cyclised RGDfK peptide. For the preparation of PAPA surfaces (lower), copolymer coatings are formed from acylamide and propargyl acrylamide which are reacted with azide modified lysine side chains on the K residue of the cyclised RDGfK(N_3_) peptide using the Cu(I) mediated azide-alkyne Huisgen cycloaddition.
